# Aqueous Extract of *Fructus Choerospondiatis* Peel Suppresses Vascular Inflammation and Alleviates Atherosclerosis via AKT/c-FOS/IL-6 Axis

**DOI:** 10.3390/nu18010021

**Published:** 2025-12-19

**Authors:** Andong Wu, Jiayi Dong, Jiankun Liu, Xueting Gong, Xueer Li, Bingbing Zhou, Ming Wan, Weixin Lv, Jiayu Qiu, Ya Zhao, Yu Fang, Jie Huang, Xiao-Li Tian

**Affiliations:** 1 Aging and Vascular Diseases, Human Aging Research Institute (HARI) and School of Life Sciences, Nanchang University, Nanchang 330031, China; andongwu@ncu.edu.cn (A.W.); jiayidong@email.ncu.edu.cn (J.D.); liujiankun0625@email.ncu.edu.cn (J.L.); gongxueting@email.ncu.edu.cn (X.G.); lixueer@email.ncu.edu.cn (X.L.); 367900220011@email.ncu.edu.cn (B.Z.); mingwan@email.ncu.edu.cn (M.W.); weixinlv@email.ncu.edu.cn (W.L.); qiujiayu@email.ncu.edu.cn (J.Q.); yazhao@email.ncu.edu.cn (Y.Z.); 405600230076@email.ncu.edu.cn (Y.F.); 2 Jiangxi Key Laboratory of Human Aging, Nanchang University, Nanchang 330031, China; 3 School of Public Health and Emergency Management, Southern University of Science and Technology, Shenzhen 518055, China; huangj@sustech.edu.cn

**Keywords:** *Fructus Choerospondiatis*, atherosclerosis, inflammation, AKT, c-FOS

## Abstract

**Background**: Atherosclerosis is the pathological basis for lethal cardio-cerebral vascular diseases, such as coronary artery disease and stroke. *Fructus Choerospondiatis* (FC) has demonstrated cardiac protective effects in multiple ethnomedicine. Whether these protective effects are attributed to the prevention of vascular atherosclerosis, however, remains unknown. We aim to examine the anti-atherosclerotic effect of FC aqueous extract and elucidate the underlying mechanism. **Methods**: FC was separated into peel and pulp, and the aqueous extract was obtained separately by boiling in water to mimic decocting. Atherosclerosis model was established in ApoE^−/−^ mice fed with a high-fat diet, and histological analysis were utilized to evaluate the development of atherosclerosis. Various inflammatory models were constructed in mice to evaluate the anti-inflammatory effect of FC extract systemically, including acute local inflammation induced by traumatic injury (ear/foot swelling), acute systemic inflammation triggered by pathogenic infection (LPS- and POLY (I:C)-induced), as well as chronic inflammatory conditions associated with oxidative stress (D-galactose-induced), metabolic disorder (db/db mice), and aging. LC-MS and network pharmacology identified bioactive components and targets. Western blotting, ELISA, qPCR, and immunofluorescence were utilized to analyze the key genes involved in the mechanisms. **Results**: FC peel extract reduced serum IL-6 level, atherosclerotic plaque area, and macrophage content in the plaque, while pulp extract showed no protective effects. Peel extract exhibits anti-inflammatory effects in all models. The integrative application of LC-MS and network pharmacology identified ellagic acid as the major bioactive component and AKT as its target protein. Mechanistically, FC peel extract inhibits AKT phosphorylation, suppresses c-FOS expression and nuclear translocation, reduces IL-6 transcription and inflammation, and thus alleviates atherosclerosis. **Conclusions**: FC peel aqueous extract exerts anti-atherosclerotic effect by inhibiting inflammation through AKT/c-FOS/IL-6 axis. This study provides novel insights into the protective effects against atherosclerosis of FC peel and highlights its potential application in the prevention and treatment of coronary artery diseases.

## 1. Introduction

Atherosclerosis, as the primary pathological basis of various cardio-cerebral vascular diseases, involves complex pathophysiological processes [1,2,3]. It is generally accepted that inflammation plays a central role in atherosclerosis development [4,5,6]. At the early stage of atherosclerosis, with endothelial activation, circulating monocytes adhere to endothelial cells and migrate into the subendothelial space under chemokine guidance, differentiate into macrophages, and uptake oxidized low-density lipoprotein, ultimately converting into foam cells [7,8,9]. This process is accompanied by a dysregulated dynamic balance between pro-inflammatory T cell subsets secreting cytokines and anti-inflammatory Treg cells, further exacerbating local inflammation [10,11]. The activation of inflammasome promotes maturation and release of pro-inflammatory cytokines (e.g., IL-1β, IL-6), establishing a positive feedback loop on amplification of inflammation [12,13,14]. The secreted inflammatory factors further promote the development of atherosclerosis [15].

*Choerospondias axillaris* is a type of plant distributed in East Asia and South Asia. Its dried ripe fruit (*Fructus Choerospondiatis*, FC) can be eaten directly as a fruit or processed and used as a seasoning or food ingredient for consumption. Also, FC is used in ethnomedicine such as Tibetan and Mongolian medicine to relieve the clinical manifestations related to coronary artery disease, such as chest pain, palpitation, and dyspnea [16]. FC exhibits multiple pharmacological properties, including anti-oxidant [17], anti-tumor [18], and anti-local inflammation activities [19]. In the heart damage related to coronary artery disease, FC shows beneficial effects on myocardial damage caused by ischemia and ischemia–reperfusion. The isolated total flavonoids of FC ameliorated cardiac dysfunction and reduced myocardial fibrosis in myocardial infarction rat model [20]. In ischemia–reperfusion injury rat model, total flavonoids of FC also reduced heart pathologic lesion and ameliorated cardiac dysfunction [21]. Citric acid and L-malic acid, which are the main organic acid components of FC, reduced myocardial infarct size in myocardial ischemia/reperfusion injury rat model [22]. Previous studies primarily focused on FC’s protective effects against myocardial injury, which arises as a pathological consequence of coronary artery disease, especially after the complete blockage of blood flow caused by the formation of blood clot. However, the protective effect of FC on atherosclerosis, the underlying pathological basis of coronary artery disease, has been ignored all along. Moreover, the content of active molecules and the bioactivities differs between the peel and pulp [23]. This suggests that it is necessary to study the peel and pulp of FC separately to provide precise insights into the health benefits of FC.

This study aims to investigate the protective effects of FC on atherosclerosis, as well as to explore its underlying mechanisms. We prepared the water extract of FC pulp and peel separately and evaluated their efficacy against atherosclerosis in ApoE^−/−^ mice fed with high-fat diet and multiple inflammatory models. Subsequently, LC-MS was utilized to screen the differential components between the pulp and peel extract, and network pharmacology analysis was performed to find the potential targets. Finally, involved mechanisms were investigated at cellular level.

## 2. Materials and Methods

### 2.1. Materials

Lipopolysaccharide (LPS) and D-galactose (D-gal) were purchased from Sigma-Aldrich Co. (St. Louis, MO, USA). Polyinosinic-polycytidylic acid (POLY (I:C)) was obtained from MedChemExpress (MCE) (Monmouth Junction, NJ, USA). ELISA kits for detecting IL-1β, IL-6, IL-10, TNF-α, and TGF-β (human and mouse) were acquired from Proteintech (Wuhan, China). Antibodies against human Phospho-AKT (*p*-AKT), AKT, and GAPDH were sourced from Cell Signaling Technology (Beverly, MA, USA), while the human c-FOS antibody was purchased from Abcam (Cambridge, UK). RNA and protein extraction reagents were obtained from Nanchang BoMingsai Biotechnology Co., Ltd. (Nanchang, China). Key PCR reagents were procured from Vazyme Biotech Co., Ltd. (Nanjing, China).

### 2.2. Water Extract of FC

FC was harvested from Yangmei Town, Chongyi County, Ganzhou, Jiangxi, China, with the coordinates of 114.48778° N and 25.67237° E. The collection date was 15 November 2022. The peel and pulp of the fresh FC were separated and dried in oven (CIMO medical instrument Co., LTD., Shanghai, China) at 60 °C and stored in a cool place at room temperature. The ratio of dried peel or pulp to water was 1:10, boiled at 100 °C for 30 min. Then, the extract was separated from the pulp or peel by filtering through sterilized gauze, and subjected to rotary evaporation (Huxi industry Co., LTD., Shanghai, China) at 80 °C until the final volume is one-tenth of the initial volume, namely, water extract of FC, which was used in subsequent experiments.

### 2.3. Animals

#### 2.3.1. Mouse Housing Conditions

Animal experiments were approved by the Animal Ethics Committee of Nanchang University (NCULAE-20221130017, 26 December 2019). All animals received humane care in compliance with the Principles of Laboratory Animal Care formulated by the National Society for Medical Research and the Guide for the Care and Use of Laboratory Animals. All animals were male C57BL/6J mice and were purchased from GemPharmatech Co., Ltd. The temperature of the mouse feeding room is maintained at a constant range of 22–25 °C, the lighting is alternated between light and dark for 12 h, and the mice eat freely. In order to ensure a clean and suitable feeding environment for mice, the cages are cleaned every week, and the number of mice in each cage does not exceed 5. Oral gavage was selected as the administration route for *Choerospondias axillaris* water extract to mimic human oral consumption and ensure controlled dosing. Anesthesia was induced using pentobarbital sodium (50 mg/kg IP) in a Class II biosafety cabinet, followed by sampling procedures.

#### 2.3.2. Study Design

Eight-week-old wild-type C57BL/6J male mice were used in this study, except for special instructions. Mice were divided into four groups: no-treatment control (NC), distilled water (H_2_O), water extract of pulp (Pulp), and water extract of peel (Peel). The NC group was fed a standard diet and distilled water without additional treatment. The H_2_O group was fed a standard diet and distilled water, subjected to model induction as required, and administered distilled water by gavage daily (50 μL/10 g body weight). The Pulp and Peel groups were fed a standard diet and distilled water, subjected to model induction, and administered the respective water extracts by gavage daily (50 μL/10 g body weight). Detailed information regarding the grouping of mice and the number of mice in each group is provided in Table S2.

#### 2.3.3. Foot Swelling Model

After 14 days of gavage, 20 μL of carrageenan was injected into the left hind paw subcutaneous tissue to induce inflammation, while an equal volume of saline was injected into the right hind paw as a control. Six hours post-injection, the mice were euthanized, and their paws were excised at the ankle for weight measurement. Swelling degree was calculated as (left paw weight − right paw weight)/right paw weight. Paw tissue homogenates were prepared, and IL-6 levels were measured by ELISA.

#### 2.3.4. Ear Swelling Model

After 14 days of gavage, 20 μL of xylene was applied evenly to the left ear (both inner and outer surfaces) to induce inflammation, while saline was applied to the right ear as a control. Six hours later, the mice were euthanized, and their ears were excised at the base for weight measurement. Swelling degree was calculated as (left ear weight − right ear weight)/right ear weight. Ear tissue homogenates were analyzed for IL-6 levels by ELISA.

#### 2.3.5. Bacterial Inflammation Model

After 14 days of gavage, mice were intraperitoneally injected with 5 mg/kg LPS. Twelve hours later, serum was collected, and IL-6 levels were measured by ELISA.

#### 2.3.6. Viral Inflammation Model

After 14 days of gavage, mice were subcutaneously injected with 30 mg/kg POLY (I:C). Twelve hours later, serum was collected, and IL-6 levels were analyzed by ELISA.

#### 2.3.7. Oxidative Stress-Induced Inflammation Model

The H_2_O, Pulp, and Peel groups were intraperitoneally injected with 100 mg/kg D-galactose (D-gal) daily for 6 weeks. Afterward, gavage was administered for 14 days, and serum was collected for IL-6 measurement by ELISA.

#### 2.3.8. Chronic Disease Inflammation Model

db/db mice were used to conduct a chronic disease inflammation model. Eight-week-old wild-type C57BL/6J mice served as the NC group, while db/db mice were divided into H_2_O, Pulp, and Peel groups. The NC group received a standard diet and distilled water without intervention. The H_2_O group was fed a standard diet and distilled water and administered distilled water by daily gavage (50 μL/10 g body weight). The Pulp and Peel groups were treated similarly but received their respective water extracts. After 14 days of gavage, serum was collected for IL-6 analysis by ELISA.

#### 2.3.9. Aging-Associated Inflammation Model

Eight-week-old wild-type C57BL/6J mice served as the NC group, while 20-month-old wild-type C57BL/6J mice were divided into H_2_O, Pulp, and Peel groups. The NC group received a standard diet and distilled water without treatment. The H_2_O group was fed a standard diet and distilled water and administered distilled water by daily gavage (50 μL/10 g body weight). The Pulp and Peel groups were treated similarly but received their respective water extracts. After 14 days of gavage, serum was collected for IL-6 measurement by ELISA.

#### 2.3.10. Atherosclerosis Model

Atherosclerosis model was constructed by feeding ApoE knockout mice with a high-fat diet. Eight-week-old ApoE^−/−^ mice were divided into H_2_O, Pulp, and Peel groups and fed a high-fat diet for 8 weeks. During this period, treatment groups received daily gavage of distilled water or water extracts (50 μL/10 g body weight). After 8 weeks, serum was collected for inflammatory cytokine analysis, and the aortic tree and aortic root were harvested for frozen sectioning and staining.

### 2.4. Cell Culture

#### 2.4.1. General Cell Culture Conditions

HUVECs were isolated from umbilical cord of various donors following the protocol previously described [24]. Informed consent was obtained from all the participating parturients. The above protocol conformed to the guidelines of the 1975 Declaration of Helsinki and was approved by the Ethics Committee of Nanchang University. Primary cells were cultured in ECM (ScienCell Research Laboratories, Carlsbad, CA, USA), supplemented with 5% fetal bovine serum (FBS), 100 mg/mL streptomycin/100 U/mL penicillin, and 1% endothelial cell growth supplement (ECGS). THP-1 is a type of monocyte cell line, and in this study, it was used to evaluate endothelial-monocytes adhesion. THP-1 cells (ATCC, USA) were cultured in RPMI medium 1640 (Gibco, Grand Island, NY, USA) containing 10% FBS. Cells were cultured in a humidified incubator at 37 °C with 5% CO_2_.

#### 2.4.2. CCK8 Assay

Cell viability was analyzed by Cell Counting Kit-8 (CCK8, Beyotime, Shanghai, China) according to the manufacturer’s protocols. The cells were treated with various concentrations of Peel extract. After treatment for 24 h, 10 μL of CCK8 reagent was added to each well and then cultured for 2 h. All experiments were performed in triplicate. The absorbance was analyzed at 450 nm using a microplate reader (Bio-Rad, Hercules, CA, USA). The proliferation of cells was expressed by the absorbance.

#### 2.4.3. Bacterial Infection-Induced Inflammation Model

Cells were cultured to an appropriate density, after which the old medium was discarded, and the cells were washed with 1× PBS. The treatment group was incubated with the Peel extract for 24 h, while the control and model groups received an equal volume of purified water. After incubation, the model and treatment groups were exposed to medium containing 10 μg/mL LPS, whereas the control group received an equal volume of 1× PBS. Six hours later, IL-6 expression levels in both cells and culture medium were measured.

#### 2.4.4. Viral Infection-Induced Inflammation Model

Cells were cultured to an appropriate density, followed by the removal of the old medium and washing with 1× PBS. The treatment group was incubated with Peel extract for 24 h, while the control and model groups received an equal volume of purified water. After incubation, the model and treatment groups were exposed to medium containing 20 μg/mL POLY (I:C), whereas the control group received an equal volume of 1× PBS. Six hours later, IL-6 expression levels in both cells and culture medium were assessed.

#### 2.4.5. Endogenous Inflammation Model

Cells were cultured to an appropriate density, after which the old medium was discarded, and the cells were washed with 1× PBS. The treatment group was incubated with Peel extract for 24 h, while the control and model groups received an equal volume of purified water. After incubation, the model and treatment groups were exposed to medium containing 20 μg/L TNF-α (Peprotech, NJ, USA), whereas the control group received an equal volume of 1× PBS. Six hours later, IL-6 expression levels in both cells and culture medium were determined.

### 2.5. ELISA

Serum IL-6, IL-1β, IL-10, and TGF-β levels were quantified with ELISA kits (Proteintech, Wuhan, China) according to the manufacturer’s instructions. In brief, 96-well plates pre-coated with capture antibody were washed three times with 300 µL wash buffer per well. Then, 100 µL standards and samples were added and incubated for 2 h at 37 °C. After three additional washes, 100 µL biotinylated detection antibody was added, and plates were incubated for 1 h at 37 °C. Wells were washed again, incubated for 30 min at 37 °C in dark with 100 µL HRP-conjugated avidin, and then washed for five times. Color was developed by adding 90 µL TMB substrate for 30 min at 37 °C. The reaction was terminated with 50 µL stop solution, and absorbance was read at 450 nm (reference 570 nm).

### 2.6. Histological Analysis

The heart and aortic tree tissues were fixed with 4% paraformaldehyde for 24 h, and the clean heart and aortic tree were stripped out for frozen embedding. The aorta root samples were sliced into 7 μm thick sections and used for Oil Red O staining and Sirius red staining. Aorta tree is used for Oil Red O staining. Oil Red O staining was used to observe patch area, and Sirius red staining was used to observe patch fiber cap area. The photos were taken with a light microscope (Carl Zeiss, Jena, Germany).

For immunofluorescence analysis, the frozen sections were incubated with specific antibodies and secondary antibodies with fluorophores. The images were taken under a Zeiss LSM800 confocal microscope. The photos are quantitatively analyzed using the ImageJ software (version: 1.51j8).

### 2.7. Cell Adhesion Assay for Monocyte-Endothelial Cells

To simulate the interaction between monocytes and endothelial cells in vivo, we co-cultured THP-1 monocytes with HUVECs to evaluate their adhesion in vitro. HUVEC was pretreated with TNF-α at a final concentration of 10 ng/mL for 6 h. After TNF-α treatment, THP-1 monocytes were labeled with 10 μM BCECF-AM (Abcam, Cambridge, UK) at 37 °C for 30 min and then added to HUVEC culture dishes for co-culture for 1 h. The cells were then gently washed with PBS to remove any unadhered THP-1 cells. The BCECF-AM labeled THP-1 cells adhered to HUVECs were quantitatively observed under fluorescence microscopy.

### 2.8. Western Blot

Cells were lysed in a RIPA buffer (Bmassay, Nanchang, China) containing protease inhibitors (Bmassay, Nanchang, China). Whole cell lysates were isolated by SDS-PAGE and then transferred to PVDF membranes (Millipore, Boston, MA, USA) for pre-activation in methanol. The membrane was incubated overnight with the primary antibody at 4 °C and then incubated with either the enzyme-labeled rabbit IgG or the anti-mouse IgG (Cell Signaling Technology, Boston, MA, USA) secondary antibody, and protein levels were quantified using a chemiluminescence detection system (Tanon, Shanghai, China).

### 2.9. Quantitative RT-PCR

Total RNA was extracted by using TRIzol Reagent (Invitrogen, Carlsbad, CA, USA) and then reverse-transcribed into cDNA using random primers (Sangon, Shanghai, China) and M-MLV reverse transcriptase (Promega, Madison, WI, USA) according to the manufacturer’s guidelines. The SYBR Green Real-Time PCR Master mix (TaKaRa, Shiga, Japan) was then used for quantitative PCR (qPCR) according to the manufacturer’s protocol.

### 2.10. LC-MS Analysis

The aqueous extract was lyophilized to obtain dry powder. Then, a 70% methanol-water solution (*v*/*v*) was added and vortexed to fully re-dissolve and mix. Subsequently, samples were centrifuged at 20,000× *g* for 10 min to remove insoluble components. Finally, before sample injection for analysis, samples were filtered using a 0.22 μm membrane before loading. Chromatographic separation was performed on a C18 column (2.1 × 100 mm, 1.7 μm). The mobile phase consists of two components: (A) 0.1% (*v*/*v*) formic acid in water and (B) 0.1% (*v*/*v*) formic acid in acetonitrile. The following gradient elution program was used at a flow rate of 0.3 mL/min: from 0 to 1 min, the concentration of B was 2%; from 1 to 16 min, the concentration of B increased linearly from 2% to 50%; from 16 to 18 min, the concentration of B rose rapidly from 50% to 100%; from 18 to 20 min, the concentration of B was 100%; from 20 to 20.1 min, the concentration of B decreased rapidly from 98% to 2%; followed by a 4.9 min re-equilibration period at 2% B. The total run time was 25 min. MS detection employed electrospray ionization (ESI) in positive/negative mode with these parameters: capillary voltage 3.5 kV, source temperature 150 °C, desolvation temperature 500 °C, cone gas flow 50 L/h, and desolvation gas flow 800 L/h. Full scan data (*m*/*z* 50–1500) were acquired with 0.2 s scan time. System control and data acquisition used MassLynx 4.1, with Progenesis QI for peak alignment (5 ppm mass tolerance), normalization, and multivariate analysis (PCA, OPLS-DA). Metabolite identification combined exact mass (<5 ppm error), MS/MS fragmentation, and database matching (HMDB, METLIN). Quality control samples (pooled samples) were injected every 10 runs to monitor system stability (RSD < 30% for QC features).

### 2.11. Network Pharmacology

The 2D chemical structure of ellagic acid (CID: 5281855) was retrieved from the PubChem database in SDF format. This structure file was uploaded to the SwissTargetPrediction database (Version: 2024.01). The prediction was performed using the “Predict Targets” function with the default parameters (Homo sapiens as the organism, probability threshold > 0). The platform utilizes a combination of 2D and 3D similarity metrics against a curated library of known active compounds to generate a ranked list of potential protein targets. All predicted targets with a probability score > 0 were collected for subsequent analysis.

Disease-associated genes were collected from the GeneCards database (Version: 5.18). The keyword “atherosclerosis” was used for searching. To ensure high relevance, genes with a Relevance Score ≥ 10 (the median score of all retrieved genes) were filtered and defined as “atherosclerosis-related genes”. This thresholding strategy helps focus on genes with stronger literature-supported associations. The predicted ellagic acid targets and the high-confidence atherosclerosis-related genes were intersected to obtain common targets, presumed to be the potential therapeutic targets of ellagic acid against atherosclerosis. This list of intersection genes was uploaded to the Metascape platform (Version: v3.5.20240101) for pathway and functional enrichment analysis. The list of intersection genes was submitted to the STRING database (Version: 12.0) to construct a PPI network. All active interaction sources were selected, and disconnected nodes were hidden in the network. The resulting TSV file containing interaction data was downloaded and imported into Cytoscape software (Version: 3.10.2) for network visualization and topological analysis.

The 3D crystal structures of the core target proteins were downloaded from the RCSB Protein Data Bank (PDB). Priority was given to structures from Homo sapiens, with high resolution (<2.5 Å) and in complex with native ligands. Proteins lacking crystal structures were homology-modeled using the SWISS-MODEL server. The 3D structure of ellagic acid was obtained from PubChem (CID: 5281855) in SDF format and converted to PDBQT format using AutoDockTools (Version: 1.5.7) after adding Gasteiger charges and merging non-polar hydrogens. Protein structures were prepared for docking by removing water molecules, adding polar hydrogen atoms, assigning Kollman charges, and defining the binding pocket (centered on the co-crystallized ligand or the predicted active site). Molecular docking was performed using AutoDock Vina (Version: 1.1.2). A grid box size of 40 × 40 × 40 Å with a grid point spacing of 1.0 Å was set to encompass the entire binding site. The docking parameters were set to the default exhaustiveness value of 8. For each protein–ligand pair, the conformation with the lowest binding affinity (highest negative score, in kcal/mol) among 10 generated poses was selected for analysis and visualization using PyMOL (Version: 2.5.0).

### 2.12. Statistical Analysis

Statistical analysis was performed using GraphPad Prism 9.1.0 software (San Diego, CA, USA). Outliers were identified and removed using the ROUT method. Normality of data distribution was assessed by the Shapiro–Wilk test. Parametric or non-parametric tests were selected based on normality test results. One-way analysis of variance (ANOVA) was conducted followed by Tukey’s post hoc test for comparisons between two groups. Intergroup analyses involving two experimental conditions were performed using either unpaired Student’s *t*-test (for normally distributed data) or the Mann–Whitney U test (for non-normally distributed data). All data are presented as mean ± SEM. A probability value of *p* < 0.05 was considered statistically significant. To quantify effect strength and confidence intervals, pairwise comparisons were conducted using Hedges’ g with 95% CI. For each comparison, the pooled standard deviation was calculated using the formula for independent samples, incorporating sample sizes and variances from both groups. The 95% CI were derived using non-central t-distribution approximations to account for potential skewness in small datasets.

## 3. Results

### 3.1. Water Extract of FC Pulp and Peel Did Not Affect Baseline Indicators of Mice

**Figure 1:**
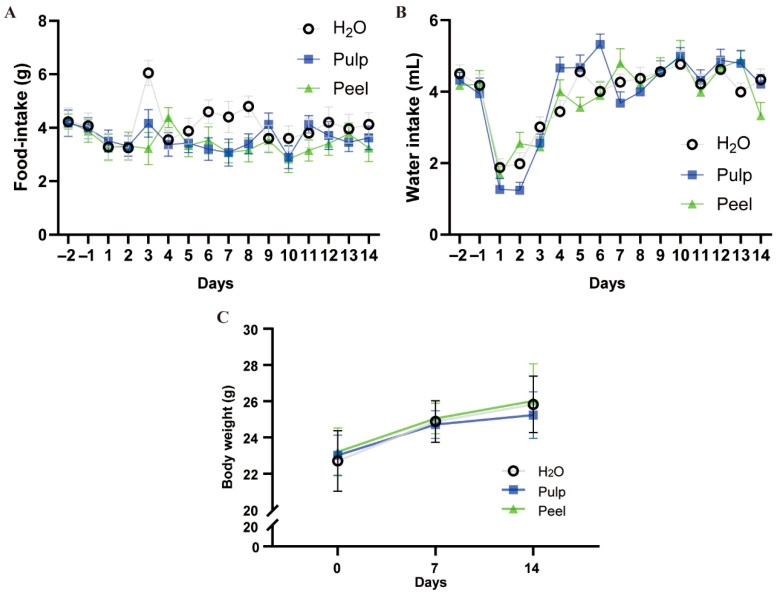
Water extract of FC pulp and peel had no effect on baseline parameters of mice. (**A**) Daily food intake, (**B**) daily water consumption, and (**C**) body weight of mice over 14 days. *n* = 6.

We separated the pulp and peel of the fruit and prepared water extracts of them. To ensure consistent intake of the water extracts for each mouse, we administered the extracts via gavage. To eliminate potential interference from gavage administration on the experimental results, we conducted a preliminary experiment: healthy eight-week-old mice were selected and subjected to continuous gavage administration of the water extracts for 14 days, with baseline indicators monitored. By monitoring daily food intake, water consumption, and body weight during the 14-day gavage period, we found that neither pulp nor peel extract gavage significantly affected the mice’s daily eating, drinking, or body weight, compared to the water gavage group (Figure 1A–C). Interestingly, the water intake of mice on the first day of gavage decreased sharply to 32–45% of that on the day before gavage (pulp extract: 1.26 ± 0.31 vs. 3.94 ± 0.44, peel extract: 1.68 ± 0.22 vs. 4.21 ± 0.38, water: 1.88 ± 0.24 vs. 4.18 ± 0.31), while gradually returned to normal level within four days. This indicate that mice need about four days to adapt to the gavage and that the pulp and peel extract had no effect on the normal life of mice.

### 3.2. Peel Extract Alleviates Atherosclerosis and Reduces Macrophage Content in Plaque

**Figure 2:**
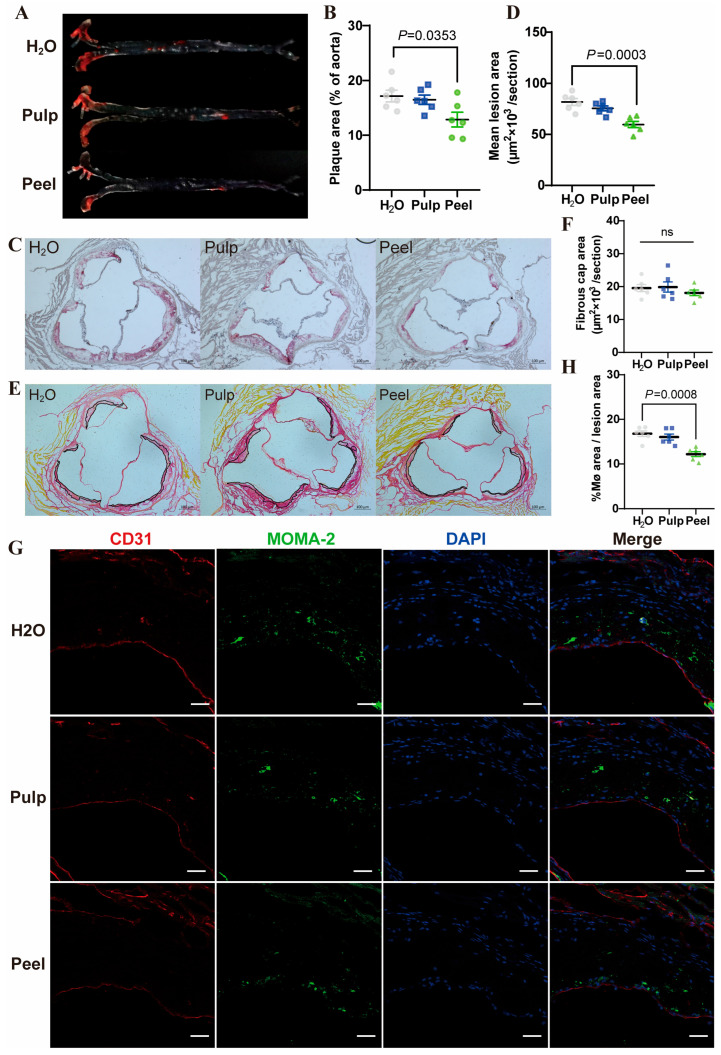
Peel extract alleviates atherosclerosis and reduces macrophage content in plaque. (**A**) Oil Red O staining of aortic trees; (**B**) quantitative analysis of plaque area in aortic trees; (**C**) Oil Red O staining of aortic root cryosections; (**D**) quantitative analysis of Oil Red O staining in aortic roots; (**E**) Sirius red staining of aortic root cryosections; (**F**) quantitative analysis of Sirius red staining in aortic roots. ns: non-significant; (**G**) immunofluorescence staining of aortic root cryosections showing CD31 (endothelial cells), MOMA-2 (macrophages), DAPI (nuclei), and merged images; (**H**) percentage area of macrophages within aortic root plaques. Biological replicates, *n* = 6. *p* < 0.05 was considered statistically significant. Scale bar: 100 μm.

Pulp and peel extract were given to high-fat diet-fed ApoE^−/−^ mice by gavage to determine whether FC extracts could play a beneficial role in atherosclerosis. The results showed that peel extract decreased atherosclerotic lesion in the whole aortic tree (Figure 2A,B, Peel vs. H_2_O Hedges’ g: −1.35, 95% CI: [−2.62, −0.07]) and aortic root (Figure 2C,D, Peel vs. H_2_O Hedges’ g: −2.58, 95% CI: [−4.18, −0.98]), while the pulp extract showed no beneficial effects. In the analysis of plaque contents, we found that peel extract significantly reduced MOMA-2 staining signal, indicating a reduced macrophage content in the plaque (Figure 2G,H). However, Sirius red staining of aortic root sections revealed that peel extract had no effect on fibrous cap (Figure 2E,F).

### 3.3. Peel Extract Reduces Inflammation and Monocyte-Endothelial Cell Adhesion

**Figure 3:**
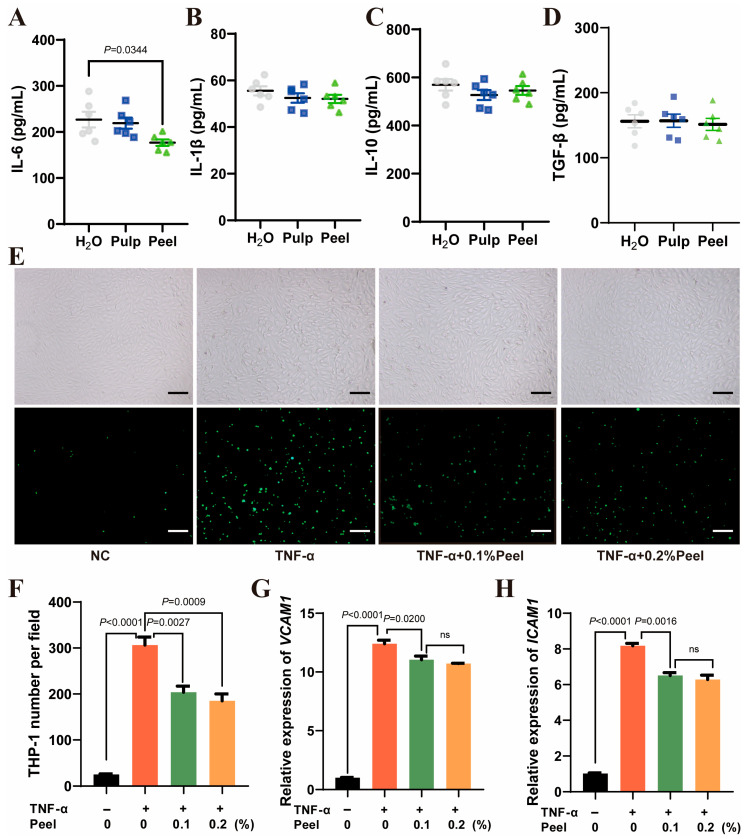
Peel extract reduces inflammation and monocyte-endothelial cell adhesion. (**A**) Serum IL-6, (**B**) IL-1β, (**C**) IL-10, and (**D**) TGF-β levels of atherosclerotic mice, *n* = 6; (**E**) bright-field images and fluorescence images of TNF-α-induced monocyte adhesion to endothelial cells. Scale bar: 100 μm; (**F**) quantitative analysis of monocyte adhesion to endothelial cells following TNF-α induction; (**G**) VCAM1 and (**H**) ICAM1 mRNA expression in TNF-α-stimulated endothelial cells, *n* = 3. *p* < 0.05 was considered statistically significant. ns: non-significant.

The adhesion of monocytes to endothelial cells, migration into the vascular wall, and transformation into macrophages are the critical steps mediated by inflammation in the early stage of atherosclerosis. Therefore, we then examined the effects of the pulp and peel extracts on the atherosclerotic mice serum inflammatory factors and monocyte-endothelial cell adhesion. As shown in Figure 3A–D, peel extract significantly reduced serum IL-6 level (Peel vs. H_2_O Hedges’ g: −1.46, 95% CI: [−2.76, −0.16]) but showed no effect on any other inflammatory factors. In the adhesion assay, peel extract significantly reduced monocyte-endothelial cell adhesion (Figure 3E,F, Peel 0.1% vs. TNF-α Hedges’ g: −3.05, 95% CI: [−5.70, −0.40]; Peel 0.2% vs. TNF-α Hedges’ g: −3.45, 95% CI: [−6.32, −0.57]) and down-regulated adhesion factors (Figure 3G,H).

### 3.4. Peel Extract Exhibits Anti-Inflammatory Effects in Multiple Inflammation Models

**Figure 4:**
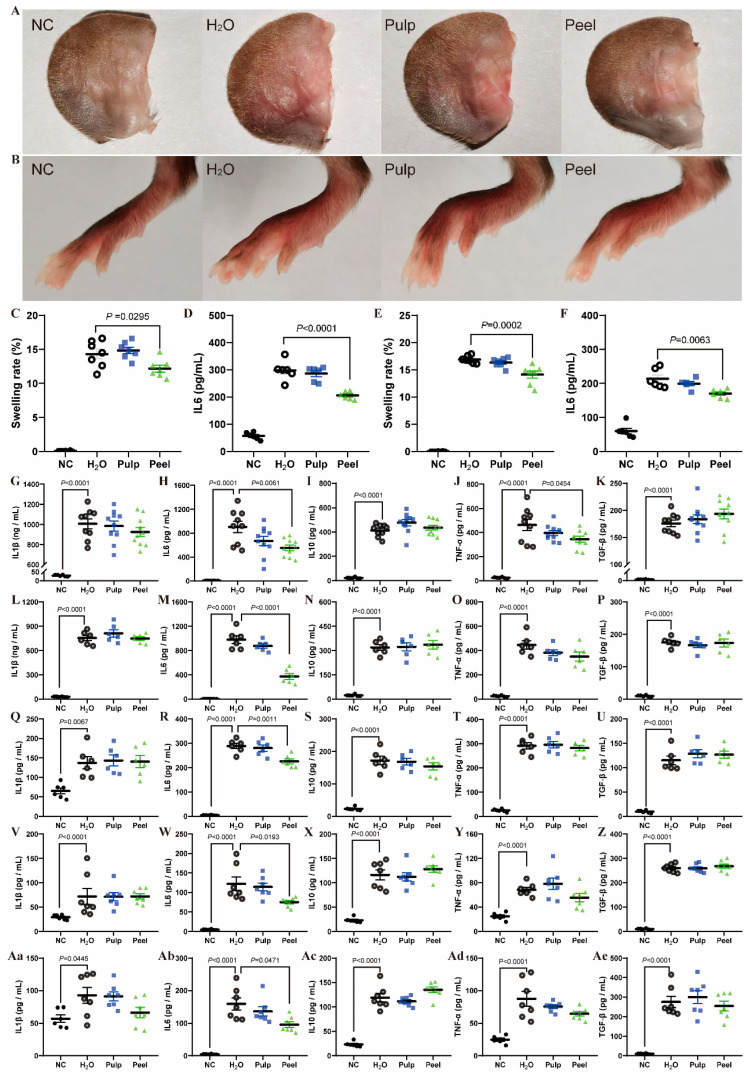
Peel extract exerts anti-inflammatory effects by down-regulating IL-6 in multiple murine inflammation models. (**A**) Representative images of ear swelling; (**B**) representative images of paw swelling; (**C**) ear swelling rate; (**D**) IL-6 levels in ear tissue; (**E**) paw swelling rate; (**F**) IL-6 levels in paw tissue; (**G**–**K**) serum levels of IL-1β, IL-6, IL-10, TNF-α, and TGF-β in LPS-induced inflammatory mice model; (**L**–**P**) serum levels of IL-1β, IL-6, IL-10, TNF-α, and TGF-β in POLY (I:C)-induced inflammatory mice model; (**Q**–**U**) serum levels of IL-1β, IL-6, IL-10, TNF-α, and TGF-β in D-gal-induced mice model; (**V**–**Z**) serum levels of IL-1β, IL-6, IL-10, TNF-α, and TGF-β in db/db mice; (**Aa**–**Ae**) serum levels of IL-1β, IL-6, IL-10, TNF-α, and TGF-β in naturally aged mice. NC group: Intragastric administration of distilled water without inflammation induction; H_2_O group: Intragastric administration of distilled water with inflammation induction; Pulp group: Intragastric administration of pulp with inflammation induction; Peel group: Intragastric administration of peel extract with inflammation induction. Biological replicates, *n* ≥ 6. *p* < 0.05 was considered statistically significant.

Given that peel extract down-regulates IL-6 expression in atherosclerosis development, we now examine its modulatory effects across multiple inflammatory models to evaluate systemic anti-inflammatory efficacy. Xylene-induced ear swelling and carrageenan-induced foot swelling represent two widely utilized models of acute injury-induced local inflammation, characterized by rapid-onset tissue damage and inflammatory mediator cascades. Peel extract significantly reduced the swelling rate and decreased IL-6 levels in both models, whereas the pulp extract showed no improvement (Figure 4A–F. Figure 4C ear swelling rate, Peel vs. H_2_O Hedges’ g: −1.20, 95% CI: [−2.36, −0.05]; Figure 4D IL-6 level, Peel vs. H_2_O Hedges’ g: −2.73, 95% CI: [−4.01, −1.45]; Figure 4E paw swelling rate, Peel vs. H_2_O Hedges’ g: −1.93, 95% CI: [−3.05, −0.81]; Figure 4F IL-6 level, Peel vs. H_2_O Hedges’ g: −1.81, 95% CI: [−3.19, −0.43]). This suggests that the peel extract exerts its anti-inflammatory effects in these two models by reducing IL-6 levels. For systemic acute inflammation models, LPS-induced and POLY (I:C)-induced models were established to simulate bacterial and viral infections, respectively. Peel extract significantly reduced serum IL-6 levels in LPS-induced model (Figure 4G–K, Figure 4H Peel vs. H_2_O Hedges’ g: −1.26, 95% CI: [−2.03, −0.49]) and POLY (I:C)-induced model (Figure 4L–P, Figure 4M Peel vs. H_2_O Hedges’ g: −3.92, 95% CI: [−5.97, −1.86]). Oxidative stress related chronic inflammation was conducted by the administration of D-gal. Peel extract was able to reduce blood IL-6 levels in this model, but pulp extract showed no effect (Figure 4Q–U, Figure 4R Peel vs. H_2_O Hedges’ g: −2.24, 95% CI: [−3.74, −0.74]). For metabolic inflammation, db/db mice is a classic type 2 diabetes mellitus (T2DM) model, with significantly elevated blood inflammatory factor levels. Peel extract significantly reduced serum IL-6 levels in db/db mice (Figure 4V–Z, Figure 4W Peel vs. H_2_O Hedges’ g: −1.37, 95% CI: [−2.66, −0.09]). Aging is accompanied by chronic inflammation; peel extract reduced serum IL-6 levels in aged mice (Figure 4Aa–Ae, Figure 4Ab Peel vs. H_2_O Hedges’ g: −1.82, 95% CI: [−3.21, −0.44]). These results demonstrate that peel extract rather than pulp extract exerts anti-inflammatory effects in multiple inflammation models, reflected by the reduction in IL-6, whereas pulp showed no anti-inflammatory activity.

### 3.5. Peel Extract Targets Aorta and Hemameba and Down-Regulates IL-6 in Endothelial Cells and Monocytes

**Figure 5:**
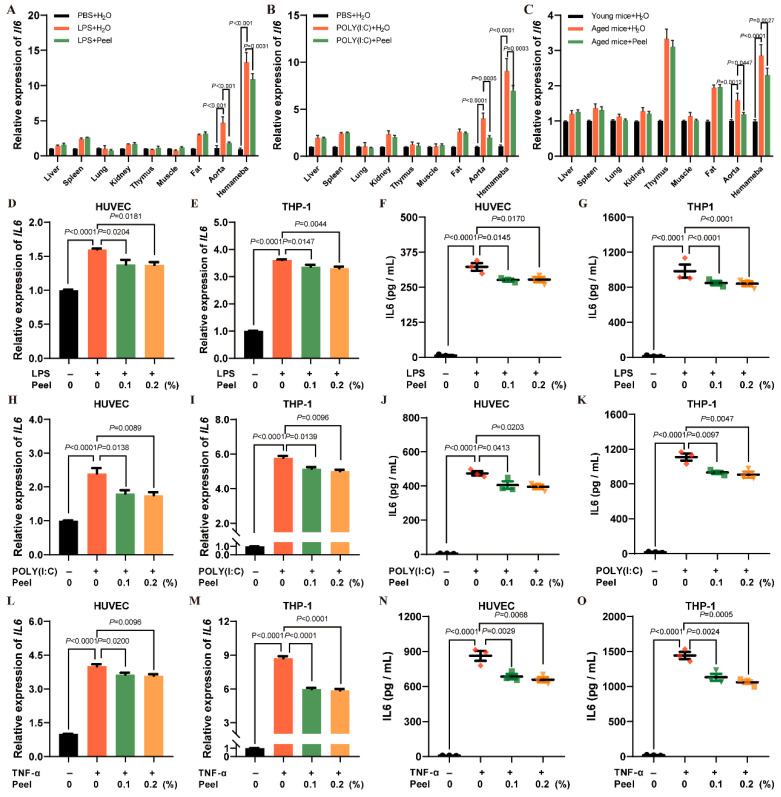
Peel extract targets aorta and hemameba and down-regulates IL-6 in endothelial cells and monocytes. (**A**) Tissue-specific IL-6 expression in LPS-induced mouse models; (**B**) tissue-specific IL-6 expression in POLY (I:C)-induced mouse models; (**C**) tissue-specific IL-6 expression in naturally aged mouse; (**D**–**G**) LPS-induced cellular inflammation model: IL-6 mRNA expression in HUVECs, IL-6 mRNA expression in THP-1 cells, IL-6 levels in HUVEC culture medium, and IL-6 levels in THP-1 culture medium; (**H**–**K**) POLY (I:C)-induced cellular inflammation model: IL-6 mRNA expression in HUVECs, IL-6 mRNA expression in THP-1 cells, IL-6 levels in HUVEC culture medium, and IL-6 levels in THP-1 culture medium; (**L**–**O**) TNF-α-induced cellular inflammation model: IL-6 mRNA expression in HUVECs, IL-6 mRNA expression in THP-1 cells, IL-6 levels in HUVEC culture medium, and IL-6 levels in THP-1 culture medium. *n* ≥ 3. *p* < 0.05 was considered statistically significant.

In the previous section, we found that the down-regulation of IL-6 was the denominator of peel extracts in multiple inflammation models. Next, we sought to determine which specific tissues and cells were affected by the peel extract. We found that IL-6 expression in the aorta and hemameba was significantly down-regulated in LPS-induced, POLY (I:C)-induced, and naturally aged mice (Figure 5A–C), suggesting that peel extract mainly targets these two tissue types. By querying The Human Protein Atlas database (https://www.proteinatlas.org/), endothelial cells and monocytes are the primary cell types responsible for IL-6 expression and secretion in these tissues. Therefore, human umbilical vein endothelial cells (HUVECs) and human monocytic leukemia cells (THP-1) were selected for subsequent cellular assay. Cellular inflammation was induced by LPS, POLY (I:C), and TNF-α. Peel extract significantly suppressed intracellular IL-6 expressions in all three inflammatory models in HUVECs and THP-1 (LPS: Figure 5D,E; POLY (I:C): Figure 5H,I; TNF-α: Figure 5L,M). In addition, the secretion of IL-6 was also significantly suppressed by peel extract (LPS: Figure 5F,G; POLY (I:C): Figure 5J,K; TNF-α: Figure 5N,O). These results indicate that peel extract exerts anti-inflammatory effects by reducing IL-6 expression and secretion in both endothelial cells and monocytes.

### 3.6. AKT Was the Potential Target of Peel Extract

**Figure 6:**
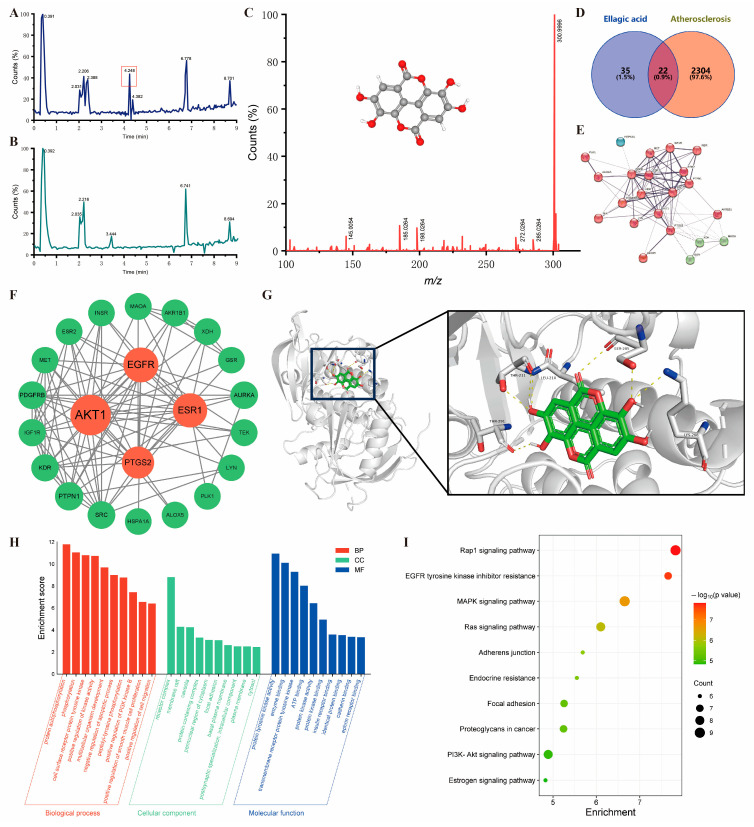
Ellagic acid is the most distinctive active component differentiating peel and pulp extracts, with AKT as its potential molecular target. (**A**) Mass spectrum of peel extract; (**B**) mass spectrum of pulp extract; (**C**) fragmentation pattern and molecular structure of ellagic acid; (**D**) Venn diagram of overlapping target genes; (**E**) protein–protein interaction (PPI) network analysis of overlapping genes; (**F**) core target screening from overlapping genes; (**G**) molecular docking of ellagic acid with AKT protein; (**H**) Gene Ontology (GO) enrichment analysis of overlapping genes; (**I**) KEGG pathway enrichment analysis of overlapping genes.

LC-MS was preformed to screen for the different contents of pulp and peel extracts. Three small molecules were identified to be significantly more abundant in the peel than in the pulp (Figure 6A,B), and the most significant one was identified to be ellagic acid (Figure 6C). Ellagic acid is a natural small molecule with known anti-inflammatory activity. Next, we intersected ellagic acid target genes from databases (https://swisstargetprediction.ch/, https://www.genecards.org/) with atherosclerosis-related genes and analyzed the interaction network of these overlapping genes, identifying AKT as the core target gene (Figure 6D–F). GO and KEGG analyses of overlapping genes revealed their association with cell adhesion-related signaling pathways (Figure 6H,I). Subsequent molecular docking results reveals that ellagic acid binds to the binding pocket region of AKT, suggesting an inhibitory effect on AKT protein phosphorylation activity (Figure 6G). These results imply that ellagic acid may be the primary active component in peel extract, with AKT as its target gene.

### 3.7. Peel Extract Exerted Anti-Inflammatory Effect Through AKT/c-FOS/IL-6 Axis

**Figure 7:**
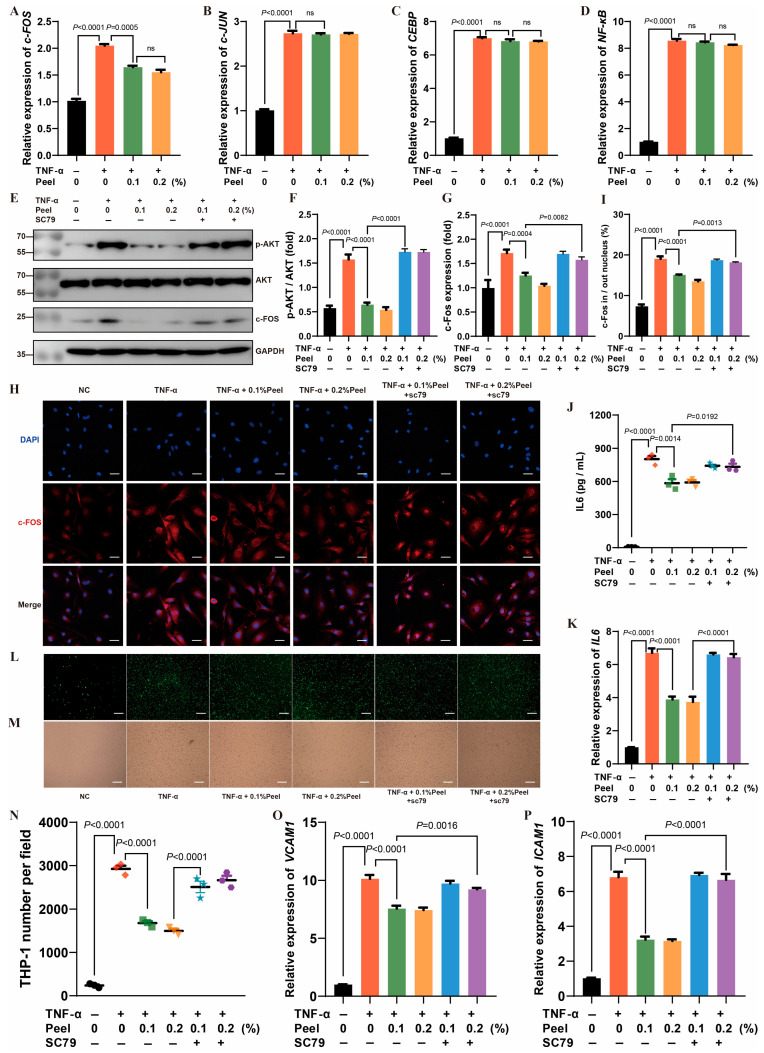
Peel extract exerted anti-inflammatory effect through AKT/c-FOS/IL-6 axis. (**A**) c-FOS mRNA expression in TNF-α-stimulated endothelial cells; (**B**) c-JUN mRNA expression in TNF-α-stimulated endothelial cells; (**C**) CEBP mRNA expression in TNF-α-stimulated endothelial cells; (**D**) NF-κB mRNA expression in TNF-α-stimulated endothelial cells; (**E**) Western blot analysis of protein expression in TNF-α-stimulated endothelial cells; (**F**) quantitative analysis of AKT phosphorylation levels; (**G**) quantitative analysis of c-FOS protein expression; (**H**) immunofluorescence co-localization of c-FOS with nuclei in TNF-α-stimulated endothelial cells. Scale bar: 20 μm; (**I**) quantitative analysis of c-FOS nuclear co-localization; (**J**) IL-6 levels in culture supernatant of TNF-α-stimulated endothelial cells; (**K**) IL-6 mRNA expression in TNF-α-stimulated endothelial cells; (**L**) FITC-labeled monocyte adhesion to TNF-α-stimulated endothelial cells. Scale bar: 100 μm; (**M**) bright-field images of monocyte adhesion to TNF-α-stimulated endothelial cells. Scale bar: 100 μm; (**N**) quantitative analysis of adherent monocytes; (**O**) VCAM1 mRNA expression in TNF-α-stimulated endothelial cells; (**P**) ICAM1 mRNA expression in TNF-α-stimulated endothelial cells. SC79 was used as a specific AKT agonist. Biological replicates, *n* = 3. *p* < 0.05 was considered statistically significant. ns: non-significant.

To explore the mechanism by which peel extract regulates IL-6 expression through AKT, we first examined the expression of IL-6 transcription factors under inflammatory condition. The mRNA expression of c-FOS was specifically inhibited by the peel extract. (Figure 7A–D). Next, we assessed the effect of peel extract on AKT phosphorylation and c-FOS protein expression. Peel extract significantly decreased AKT phosphorylation and c-FOS protein levels, and these effects were abolished by the AKT agonist SC79 (Figure 7E–G). Entering the nucleus is the prerequisite for the transcription factors to exert their functions. Immunofluorescence results demonstrated that peel extract significantly retained c-FOS protein in the cytoplasm, whereas SC79 counteracted this effect (Figure 7H,I). Concurrently, we measured IL-6 expression under inflammatory conditions and found that peel extract reduced IL-6 expression and protein levels, and this down-regulation effect can be rescued by SC79 (Figure 7J,K). Furthermore, the inhibitory effect of peel extract on the adhesion of monocytes-endothelial cells and the expression of adhesion factors was reversed by SC79. (Figure 7L–P). These results indicate that peel extract reduces IL-6 levels by inhibiting AKT phosphorylation, thereby decreasing c-FOS expression and nuclear translocation. Through this signaling pathway, peel extract exhibits anti-inflammatory effects, reduces the adhesion of monocytes to endothelial cells, and plays a role in anti-atherosclerosis.

## 4. Discussion

This study examined the anti-atherosclerotic effect of FC pulp and peel and found that the peel water-extract alleviated atherosclerotic development and reduced macrophage content in the plaque, while the pulp extract showed no protective effect. Further, the peel extract exhibited a broad-spectrum anti-inflammatory effect. The common anti-inflammatory property of the peel extract is the inhibition of IL-6 expression level. Ellagic acid was identified to be the most distinctive active component between the peel and pulp extracts, and AKT was screened as the core target gene. Peel extract was found to inhibit AKT phosphorylation and suppress both the expression and nuclear translocation of the IL-6 transcription factor c-FOS, thereby effectively inhibiting IL-6 expression.

FC was used for the treatment of coronary artery disease in multiple traditional ethnic medicines. In network pharmacology analysis, the components of FC ethanol extract regulated AMPK and PPAR signaling pathway by 65 target genes. Further cellular experimental validation proved that FC treatment decreased the expression of PPARg, thus alleviated coronary artery disease by attenuating hypoxia-reoxygenation injury [25]. In vivo rat model confirmed the protective effect of FC on myocardial ischemia–reperfusion injury [21]. In addition, FC ameliorating cardiac dysfunction and reducing myocardial fibrosis in myocardial infarction rat model [20]. These findings indicate that FC plays a beneficial role in myocardial damage caused by hypoxia resulting from coronary artery disease. Here, we provide experimental evidence that the peel extract of FC alleviates atherosclerosis. This indicates that, in addition to the protective effect on myocardial damage, FC also shows vascular protective effect, highlighting the application value of FC in the prevention of coronary artery disease and other atherosclerosis-related diseases, such as stroke, peripheral artery disease, and renovascular hypertension.

Interestingly, the peel extract showed anti-atherosclerotic and multiple anti-inflammatory effects, while the pulp extract showed none. This is most likely due to the difference in the content of bioactive small molecules in the peel and pulp. Previous studies found that the total phenolic content of peel was significantly higher than that of pulp [23], and the peel extract showed a stronger anti-inflammatory effect than the pulp extract in the xylene-induced ear edema local inflammation model [26]. Notably, we obtain the extract by using the decoction method, which is a commonly used method in traditional medicine. The milder water extraction method may not be able to fully extract the less abundant active substances present in the pulp, while the thorough alcohol extraction method used in previous studies can. This was supported by our mass spectrum result. The peel extract contains a relatively high amount of ellagic acid, but there is no such peak in the pulp extract at all.

We found that FC peel extract down-regulates IL-6 in both monocytes and endothelial cells, suppresses endothelial activation and monocyte-endothelial cell adhesion, thus alleviates atherosclerosis. Supportively, IL6 was the predicted target gene of FC in network pharmacology analysis [25]. FC was reported to exhibit anti-inflammatory effects in some local inflammation. The alcohol extract of FC down-regulated IL-6 and TNF-α in rheumatoid arthritis and osteoarthritis rat models [19] and inhibited ear edema induced by xylene. Here, we showed that FC peel extract demonstrates a broad-spectrum anti-inflammatory effect in various acute and chronic inflammatory conditions, with the down-regulation of IL-6 being the common feature of the inhibition of all types of inflammation by the FC peel extract. IL-6 plays a pivotal role as a key inflammatory factor in inflammatory responses [6,27,28]. In acute local inflammation, IL-6 enters the circulatory system, triggering systemic inflammatory response and initiating inflammatory cascade that spreads from the local site to the entire body [29]. In the inflammation caused by pathogen infection, IL-6 regulates immune cells to trigger immune responses and eliminate the pathogen, while its excessive elevation exacerbates the systemic inflammatory response, leading to vasodilation, increased permeability, hypotension, shock, and even multiple organ dysfunction syndrome [30]. In chronic inflammation, the high expression of IL6 can disrupt tissue homeostasis, cause tissue damage, and thereby lead to organ dysfunction and the progression of related chronic diseases [31]. The broad-spectrum inhibitory effect of FC on IL-6 expression indicates its role in protecting against excessive inflammatory responses and mitigating the associated damage.

LC-MS analysis found ellagic acid as the most distinctive compound in the peel extract. Ellagic acid exhibited inhibitory effects on various inflammations [32,33,34]. Ellagic acid was detected in the extract of both peel and pulp by alcohol extraction [23], potentially explaining the comparable anti-inflammatory efficacies of peel and pulp extract [26]. AKT was screened as the target of ellagic acid. Ellagic acid was reported to suppress AKT-dependent signaling cascades, with particular emphasis on the NF-κB/AKT axis, a regulator of inflammatory responses [35], and NF-κB signaling plays regulatory role in the development of atherosclerosis [36]. In this study, we found that among the four transcription factors regulating IL-6 expression, the expression levels of three (c-JUN, CEBP, and NF-κB) remained unchanged, while only c-FOS exhibited a significant downregulation following treatment with FC peel extract. This indicates that the down-regulation of IL-6 level by FC peel extract is achieved through c-FOS rather than other transcription factors, such as NF-κB, which has been emphasized in previous studies. Supportively, ellagic acid has been shown to down-regulate c-FOS expression during osteoclastogenesis, thereby mediating its protective effects against osteoporosis [37,38]. The down-regulation and inhibited nuclear translocation of c-FOS by FC peel extract are the key mechanisms by which it inhibits the transcriptional activity of c-FOS and thereby reduces the level of IL-6. This suggests that AKT/c-FOS/IL-6 is the crucial signaling cascade affected by FC peel extract for its anti-inflammatory and anti-atherosclerotic effects.

In summary, our study showed that FC peel extract, rather than pulp extract, demonstrates anti-atherosclerotic effect by inhibiting the inflammation. Peel extract down-regulates IL-6 and adhesion factors, reduces the adherence of monocytes and endothelial cells, decreases the monocyte/macrophage content within the plaque, and alleviates atherosclerosis. Furthermore, FC peel extract demonstrates multifaceted anti-inflammatory effects in various inflammatory models, including acute local inflammation induced by traumatic injury, acute systemic inflammation triggered by pathogenic infection, and chronic inflammatory conditions associated with oxidative stress, metabolic disorder, and aging-related processes. By contrast, the water extract of FC pulp showed no protective effect. The peel extract down-regulates the expression of IL-6 in all inflammatory models, by inhibiting the phosphorylation of AKT, thereby suppressing the expression and nuclear translocation of c-FOS.

The standardization of the aqueous *Choerospondias axillaris* extract provides a solid material basis for the reliability of all subsequent pharmacological predictions and mechanistic analyses. The relative deviation of the solid content in this study could be controlled within a range of <5%. Thus, we deliver a candidate extract that has undergone preliminary quality control and is ready for in-depth pharmacodynamic validation. This standardization protocol fulfills the fundamental quality requirements for herbal preparations entering pre-clinical development and establishes a requisite benchmark for future dose–response studies and formulation work. Nevertheless, full safety-quality specification, including limits for heavy metals, pesticide residues, and microbial contamination, must be appended in accordance with the Chinese Pharmacopeia or relevant international guidelines for further drug development.

The strength of this study is the demonstration that *Fructus Choerospondiatis* peel extract exerts broad-spectrum anti-inflammatory activity across multiple cellular and murine models and attenuates atherosclerosis in ApoE^−/−^ mice, thus linking general anti-inflammatory efficacy to a clinically relevant vascular disease. Beyond demonstrating the protective effects of FC Peel extract in both atherosclerotic and multiple inflammatory models, the present study quantified the magnitude of its intervention through effect-size analysis. Peel extract produced medium-to-large Hedges’ g values with 95% confidence intervals that excluded zero, indicating robust precision and reproducibility. These consistent, measurable inhibitory effects provide quantitative, high-confidence evidence that the peel extract exerts potent anti-inflammatory and anti-atherogenic actions. From a translational perspective, the observed effect sizes furnish an initial quantitative rationale for the clinical relevance of peel and its molecular targets. Although animal models differ from human disease, the magnitude and trans-model consistency of these effects highlight the therapeutic promise of modulating the AKT/c-FOS/IL-6 axis for IL-6-driven chronic inflammatory disorders such as atherosclerosis. However, there are several issues that remain unaddressed in this study: (i) determining the metabolic profile of the extract after oral administration to understand its metabolic pathways; (ii) determining the maximum tolerated dose and systemic toxicity to assess its safety; and (iii) conducting human pharmacokinetic and bioavailability studies to facilitate its clinical translation and drug development.

## 5. Conclusions

In conclusion, this study demonstrates that FC peel extract exerts atheroprotective and anti-inflammatory effects by modulating the AKT/c-FOS/IL-6 axis. Our findings provide novel insights into the clinical application of FC, especially on inflammation-related diseases. Future investigations should focus on elucidating the health benefits and mechanisms of the bioactive components and the metabolites of FC, thereby facilitating its potential therapeutic applications in disease prevention and treatment.

## Data Availability

The original contributions presented in this study are included in the Supplementary Materials. Further inquiries can be directed to the corresponding author.

## Supplementary Materials

The following supporting information can be downloaded at: https://www.mdpi.com/article/10.3390/nu18010021/s1, Table S1: Primer sequences for Real-Time PCR; Table S2: The mice used in this study; Figure S1: Western blot raw data of p-AKT in Figure 7; Figure S2: Western blot raw data of AKT in Figure 7; Figure S3: Western blot raw data of c-FOS in Figure 7; Figure S4: Western blot raw data of GAPDH in Figure 7.
