# Supplementary material for: Aqueous Extract of Fructus Choerospondiatis Peel Suppresses Vascular Inflammation and Alleviates Atherosclerosis via AKT/c-FOS/IL-6 Axis
Source: Nutrients. 2025 Dec 19;18(1):21. doi: 10.3390/nu18010021 (PMC12787844; doi:10.3390/nu18010021)

Table S1. Primer sequences for Real-Time PCR

Gene	Forward	Reverse
Human IL-6	ACTCACCTCTTCAGAACGAATTG	CCATCTTTGGAAGGTTCAGGTTG
Mouse IL-6	TAGTCCTTCCTACCCCAATTTCC	TTGGTCCTTAGCCACTCCTTC
Human c-FOS	CCGGGGATAGCCTCTCTTACT	CCAGGTCCGTGCAGAAGTC
Human c-JUN	TCCAAGTGCCGAAAAAGGAAG	CGAGTTCTGAGCTTTCAAGGT
Human CEBP	GCCTGCTATAGGCTGGGCTT	GCTGACCCACGACCTAGCTT
Human NF-κB	AACAGAGAGGATTTCGTTTCCG	TTTGACCTGAGGGTAAGACTTCT
Human VCAM1	AGTTGGGGATTCGGTTGTTCT	CCCCTCATTCCTTACCACCC
Human ICAM1	GTGATGCTCAGGTATCCATCCA	CACAGTTCTCAAAGCACAGCG

Table S2. The mice used in this study.

Models	Mice information	Group	Mice number	Body weight (g, mean±SD)	P value
Foot Swelling Model	C57BL/6J, 8-week old, male, wild type	NC	7	24.49±0.6149	/
		H_2O	7	24.54±0.5884	0.9965
		Pulp	7	24.70±0.6351	0.8605
		Peel	7	24.61±0.6768	0.9634
Ear Swelling Model	C57BL/6J, 8-week old, male, wild type	NC	7	24.53±0.6525	/
		H_2O	7	24.36±0.5855	0.9105
		Pulp	7	24.56±0.6161	0.9994
		Peel	7	24.73±0.5469	0.8681
Bacterial Inflammation Model	C57BL/6J, 8-week old, male, wild type	NC	6	24.43±0.6683	/
		H_2O	9	24.57±0.6500	0.9514
		Pulp	10	24.74±0.6769	0.6444
		Peel	10	24.57±0.5618	0.9451
Viral Inflammation Model	C57BL/6J, 8-week old, male, wild type	NC	6	24.77±0.8091	/
		H_2O	6	24.82±0.7985	0.9990
		Pulp	6	24.80±0.6957	0.9997
		Peel	6	24.63±0.8287	0.9824
Oxidative Stress-Induced Inflammation Model	C57BL/6J, 8-week old, male, wild type	NC	6	26.15±0.7994	/
		H_2O	6	25.62±0.7705	0.5188
		Pulp	6	25.20±0.8899	0.1216
		Peel	6	25.27±0.6772	0.1590
Chronic Disease Inflammation Model	C57BL/6J, 8-week old, male, wild type	NC	6	26.15±0.7994	<0.0001
	C57BLKS/J, 8-week old, male, db/db	H_2O	7	47.46±5.223	/
		Pulp	7	47.11±4.699	0.9988
		Peel	7	46.44±7.719	0.9706
Aging-Associated Inflammation Model	C57BL/6J, 8-week old, male, wild type	NC	6	26.15±0.7994	<0.0001
	C57BL/6J, 20-month old, male, wild type	H_2O	7	34.47±1.792	/
		Pulp	7	35.41±2.186	0.6411
		Peel	7	35.01±1.907	0.8952
Atherosclerosis Model	C57BL/6J, 8-week old, male, $ApoE^{-/-}$	H_2O	6	25.38±1.015	/
		Pulp	6	25.72±1.019	0.7902
		Peel	6	25.57±0.9626	0.9293

Fig S1. Western blot raw data of p-AKT in Fig 7.

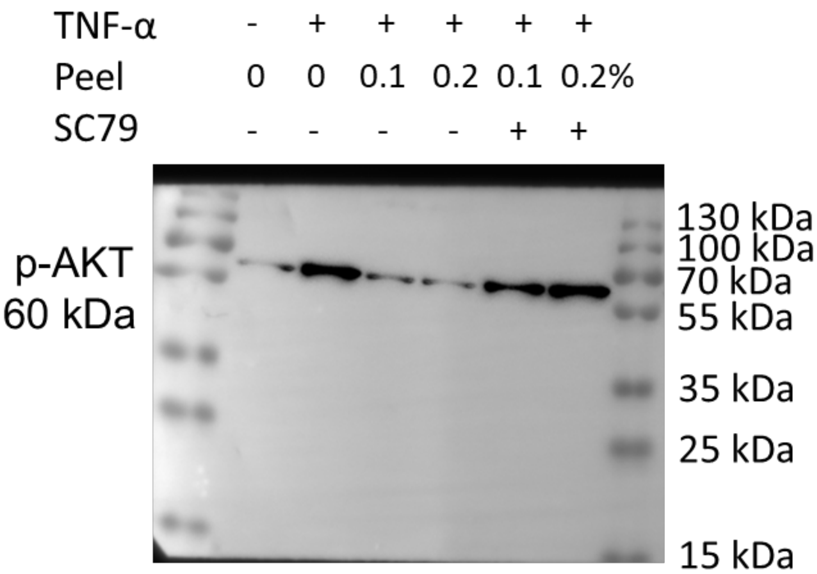

Fig S2. Western blot raw data of AKT in Fig 7.

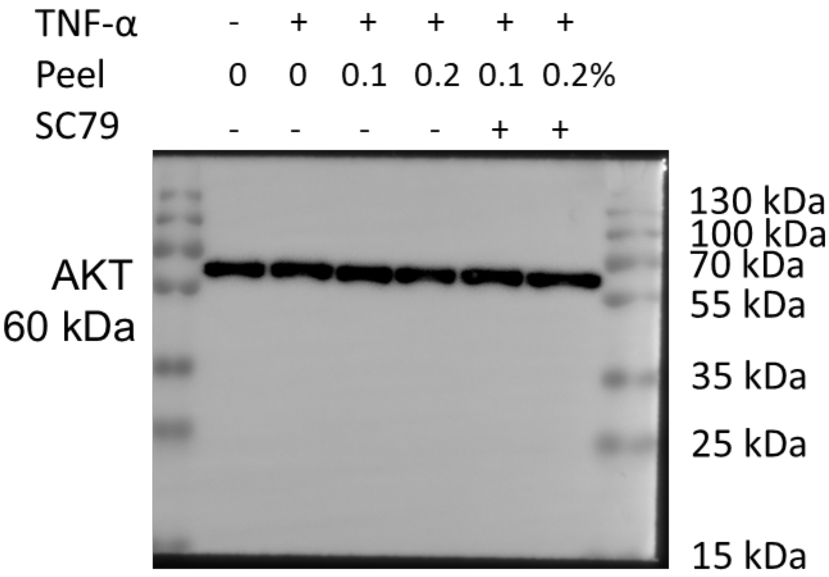

Fig S3. Western blot raw data of c-FOS in Fig 7.

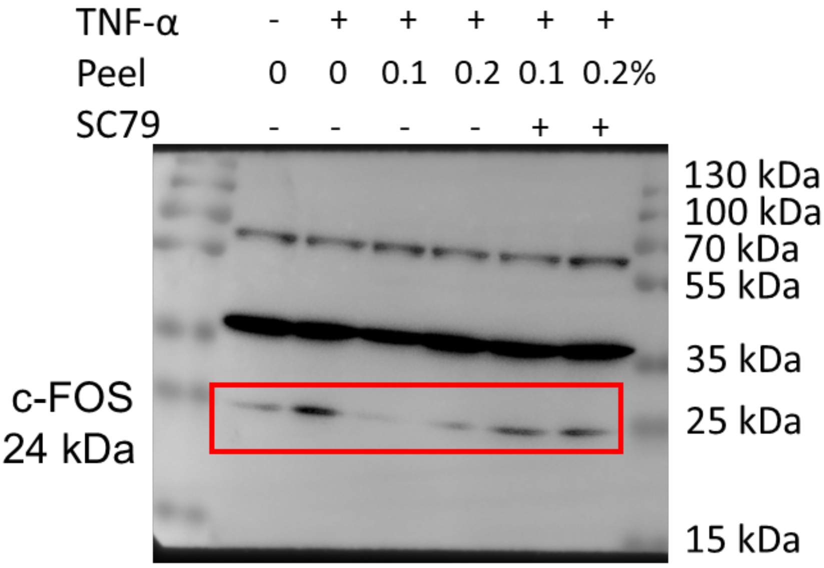

Fig S4. Western blot raw data of GAPDH in Fig 7.

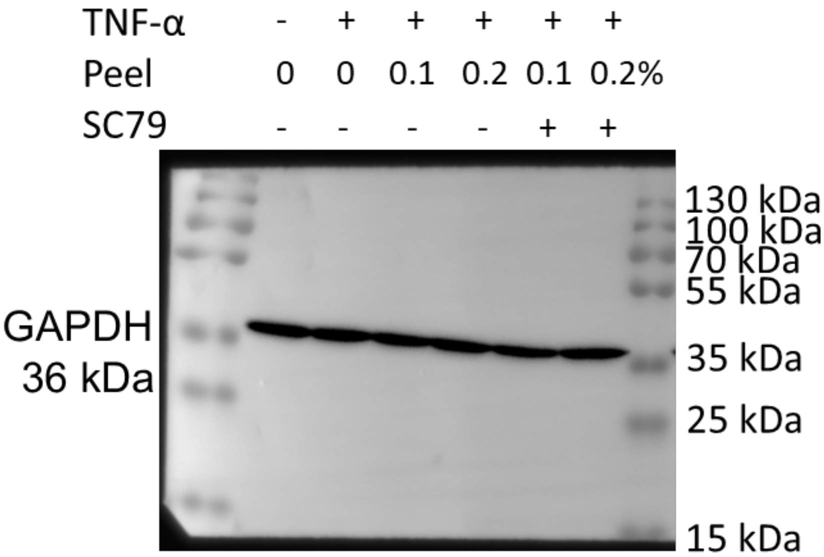

Supplement: Supplementary file 1 [file nutrients-18-00021-s001.zip › Supplementary information revised.pdf]
