# Supplementary figures and images for: Aqueous Extract of Fructus Choerospondiatis Peel Suppresses Vascular Inflammation and Alleviates Atherosclerosis via AKT/c-FOS/IL-6 Axis
Source: Nutrients. 2025 Dec 19;18(1):21. doi: 10.3390/nu18010021 (PMC12787844; doi:10.3390/nu18010021)

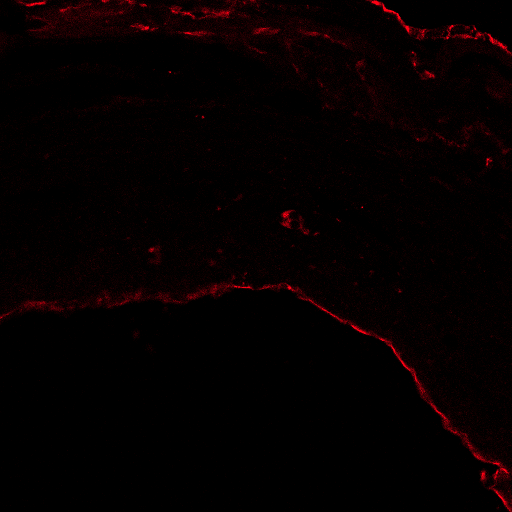

Supplement: Supplementary file 1 [file nutrients-18-00021-s001.zip › Raw image of Fig 2G and 3E/Fig 2G/Fig 2G-1.tif]

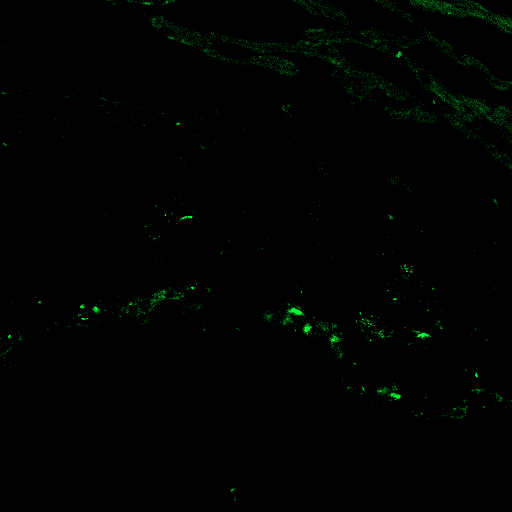

Supplement: Supplementary file 1 [file nutrients-18-00021-s001.zip › Raw image of Fig 2G and 3E/Fig 2G/Fig 2G-10.tif]

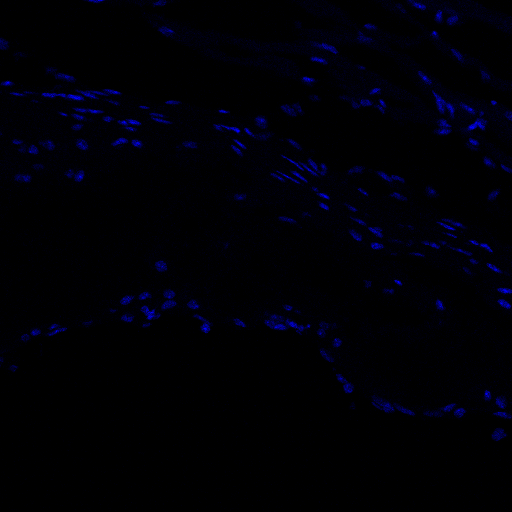

Supplement: Supplementary file 1 [file nutrients-18-00021-s001.zip › Raw image of Fig 2G and 3E/Fig 2G/Fig 2G-11.tif]

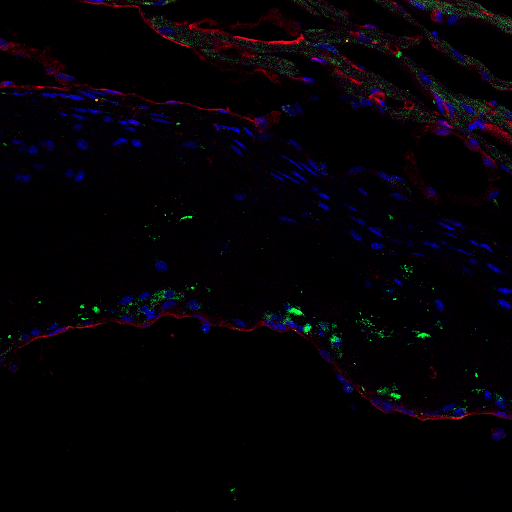

Supplement: Supplementary file 1 [file nutrients-18-00021-s001.zip › Raw image of Fig 2G and 3E/Fig 2G/Fig 2G-12.tif]

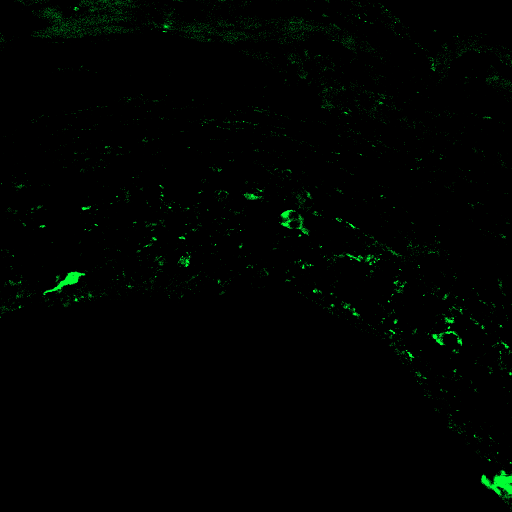

Supplement: Supplementary file 1 [file nutrients-18-00021-s001.zip › Raw image of Fig 2G and 3E/Fig 2G/Fig 2G-2.tif]

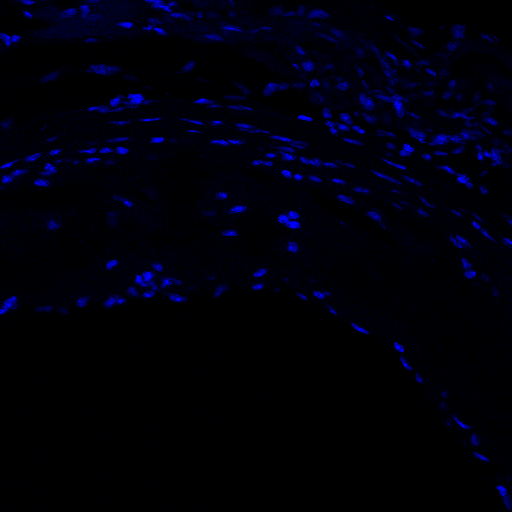

Supplement: Supplementary file 1 [file nutrients-18-00021-s001.zip › Raw image of Fig 2G and 3E/Fig 2G/Fig 2G-3.tif]

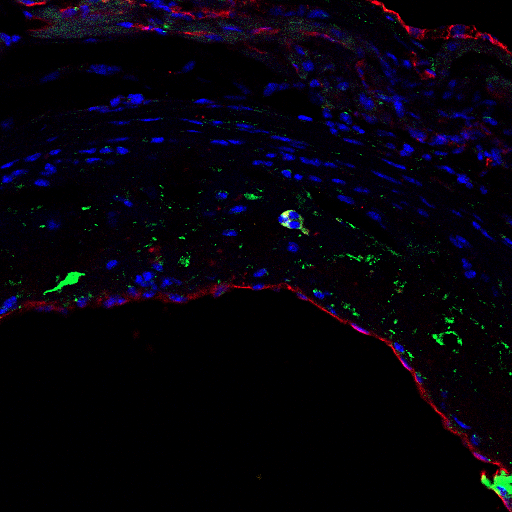

Supplement: Supplementary file 1 [file nutrients-18-00021-s001.zip › Raw image of Fig 2G and 3E/Fig 2G/Fig 2G-4.tif]

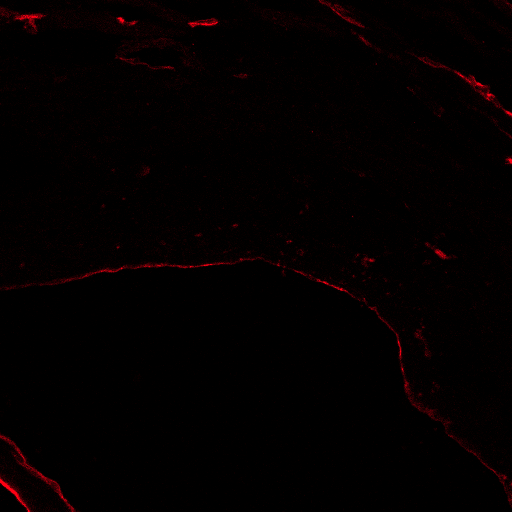

Supplement: Supplementary file 1 [file nutrients-18-00021-s001.zip › Raw image of Fig 2G and 3E/Fig 2G/Fig 2G-5.tif]

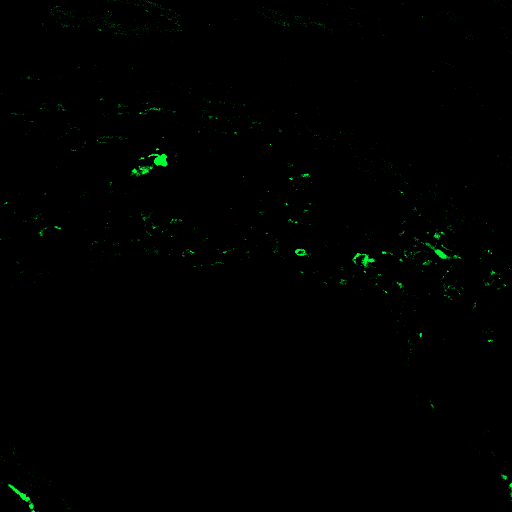

Supplement: Supplementary file 1 [file nutrients-18-00021-s001.zip › Raw image of Fig 2G and 3E/Fig 2G/Fig 2G-6.tif]

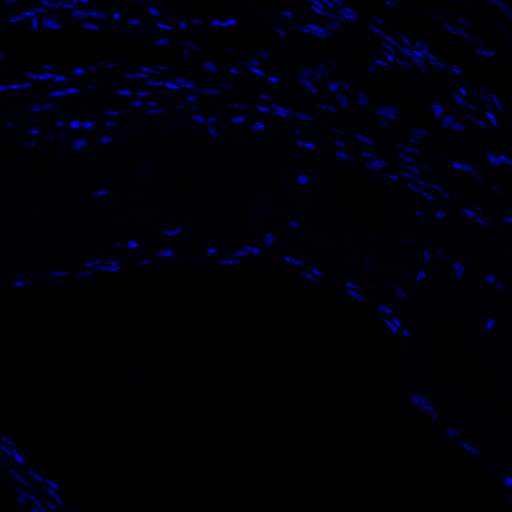

Supplement: Supplementary file 1 [file nutrients-18-00021-s001.zip › Raw image of Fig 2G and 3E/Fig 2G/Fig 2G-7.tif]

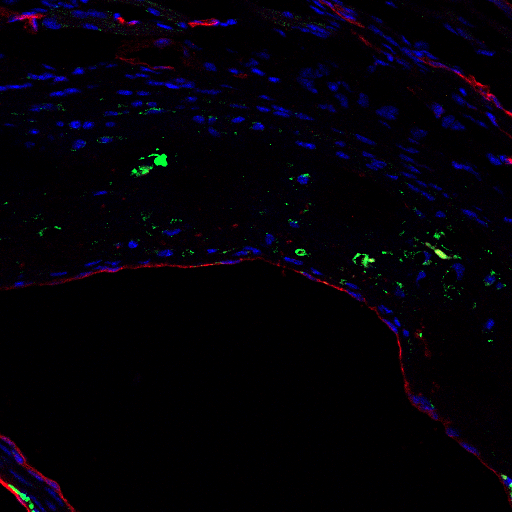

Supplement: Supplementary file 1 [file nutrients-18-00021-s001.zip › Raw image of Fig 2G and 3E/Fig 2G/Fig 2G-8.tif]

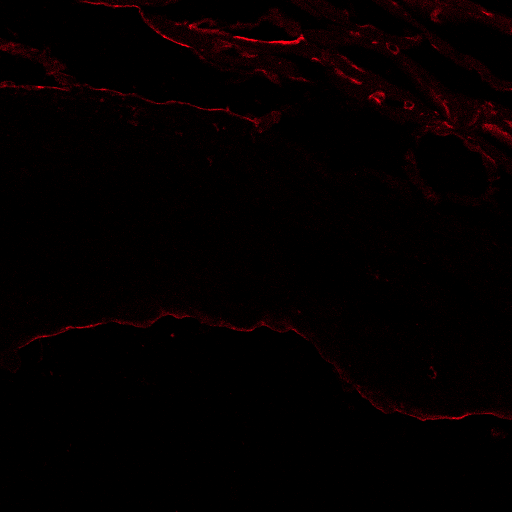

Supplement: Supplementary file 1 [file nutrients-18-00021-s001.zip › Raw image of Fig 2G and 3E/Fig 2G/Fig 2G-9.tif]

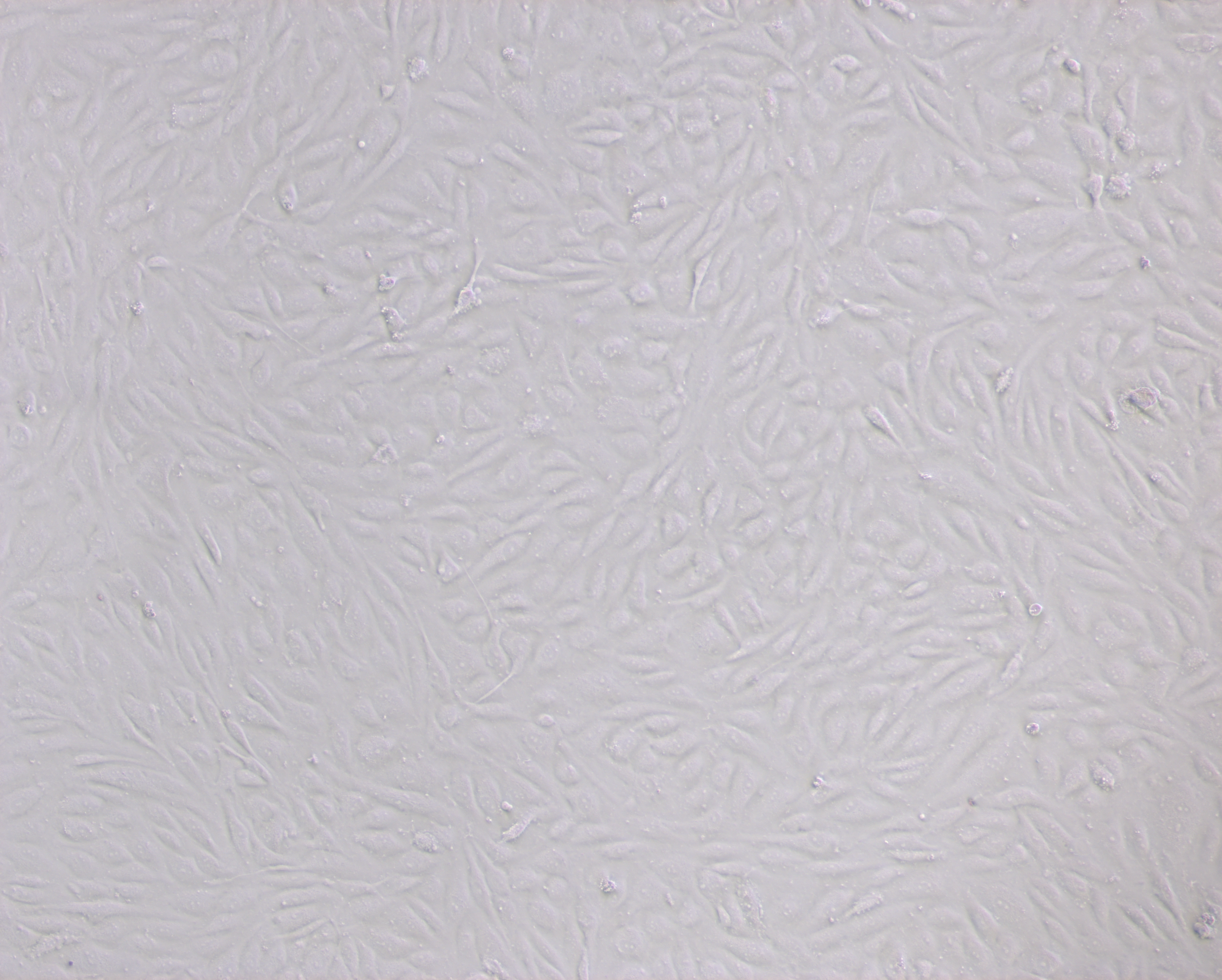

Supplement: Supplementary file 1 [file nutrients-18-00021-s001.zip › Raw image of Fig 2G and 3E/Fig 3E/Fig 3E -1.tif]

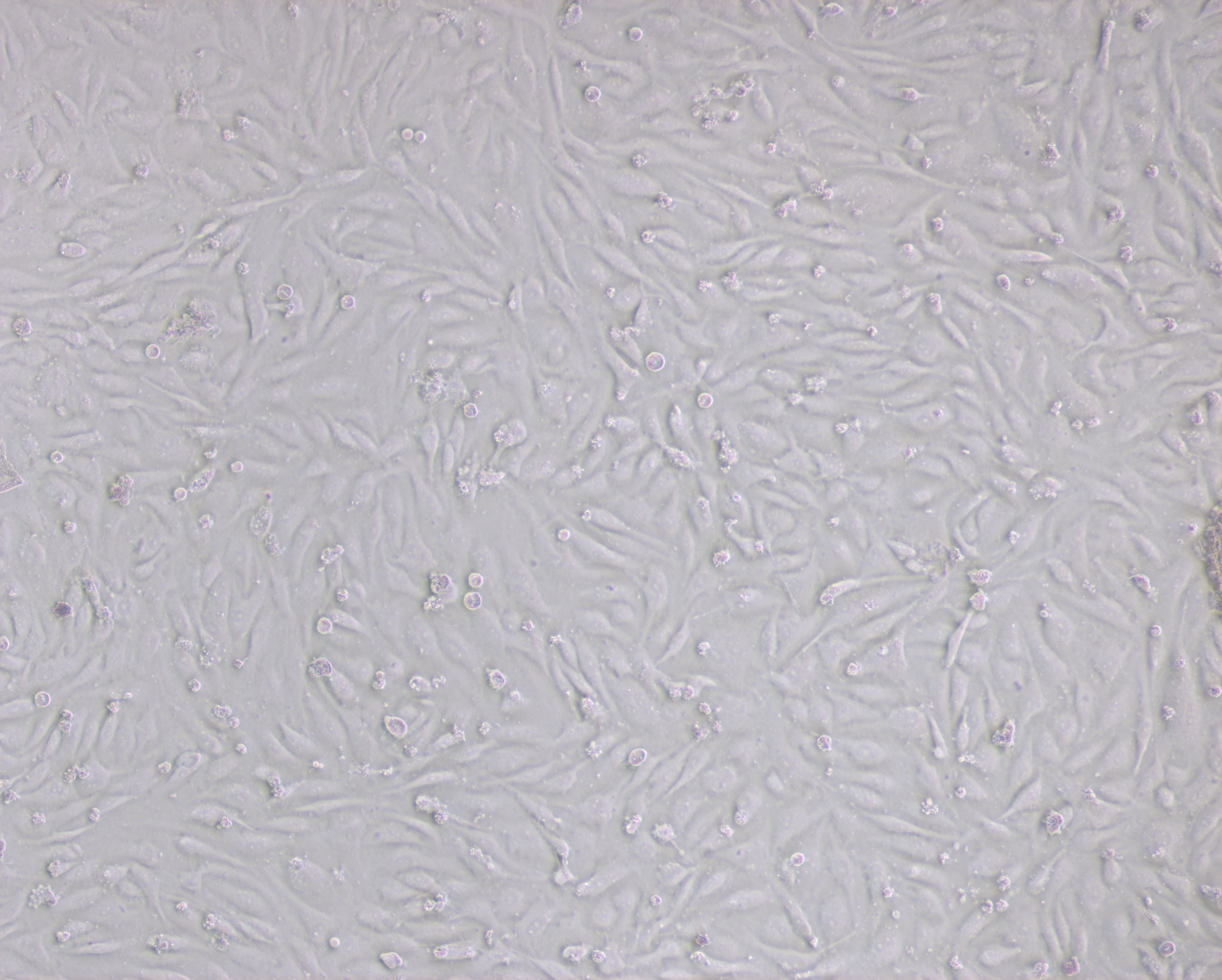

Supplement: Supplementary file 1 [file nutrients-18-00021-s001.zip › Raw image of Fig 2G and 3E/Fig 3E/Fig 3E -2.tif]

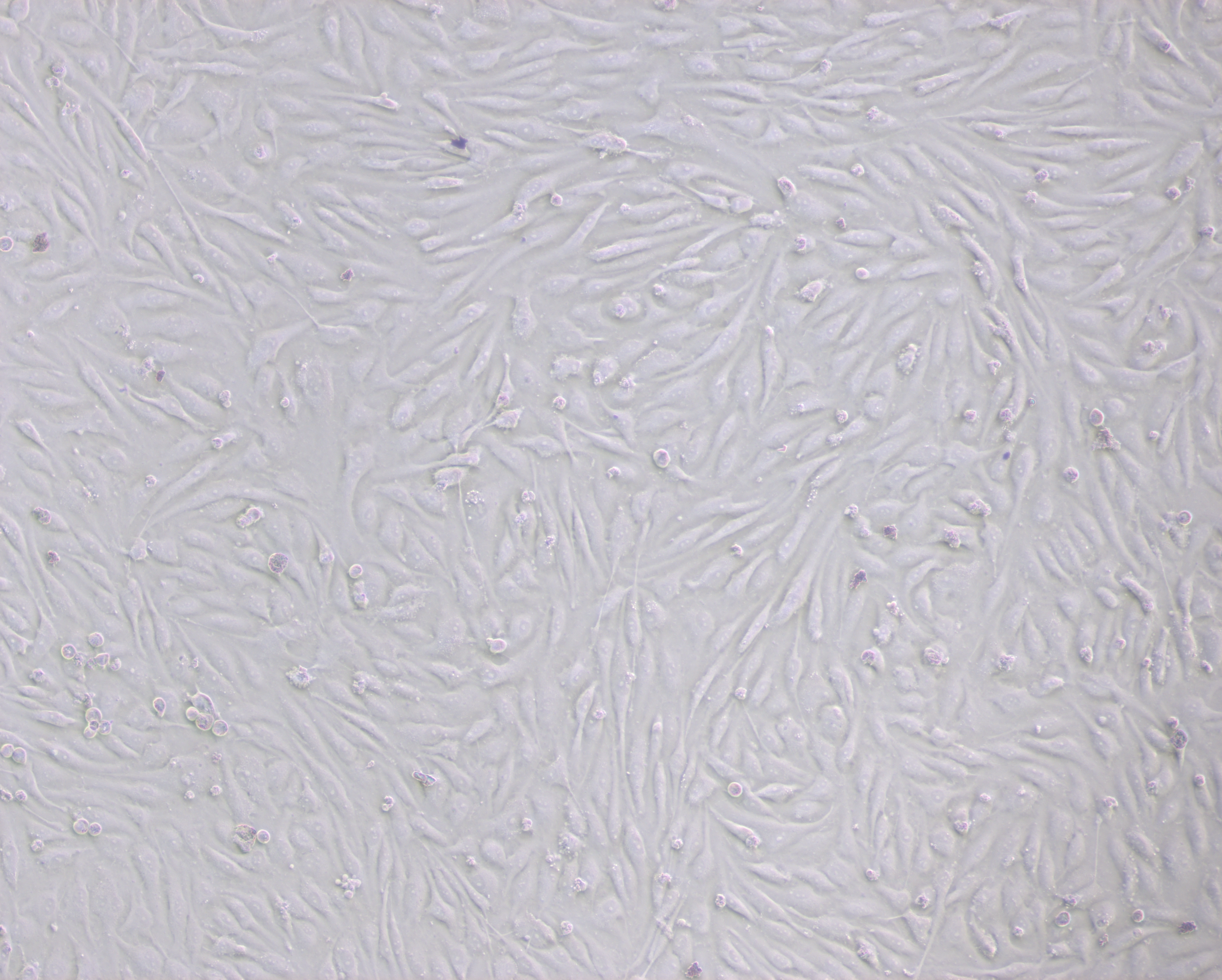

Supplement: Supplementary file 1 [file nutrients-18-00021-s001.zip › Raw image of Fig 2G and 3E/Fig 3E/Fig 3E -3.tif]

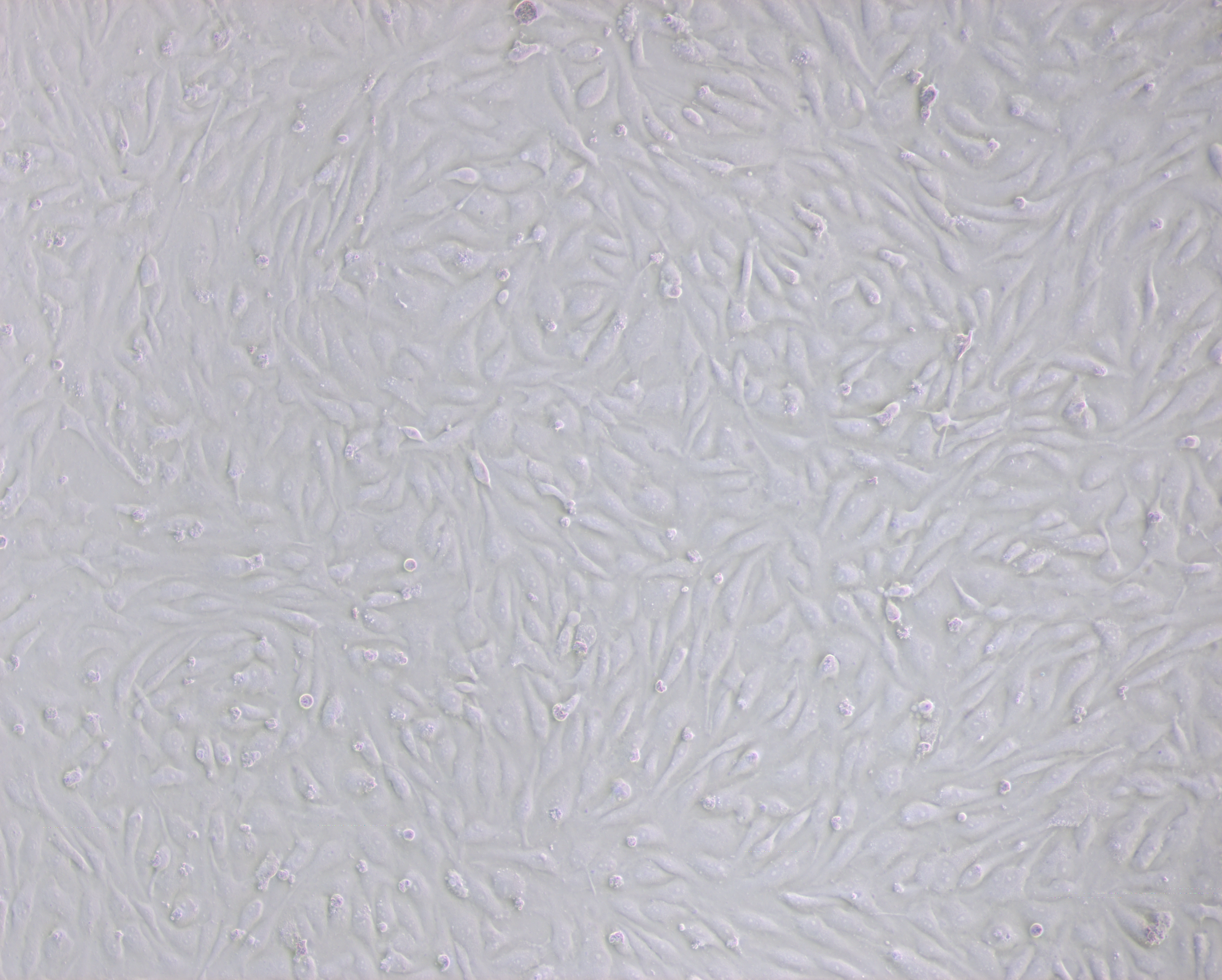

Supplement: Supplementary file 1 [file nutrients-18-00021-s001.zip › Raw image of Fig 2G and 3E/Fig 3E/Fig 3E -4.tif]

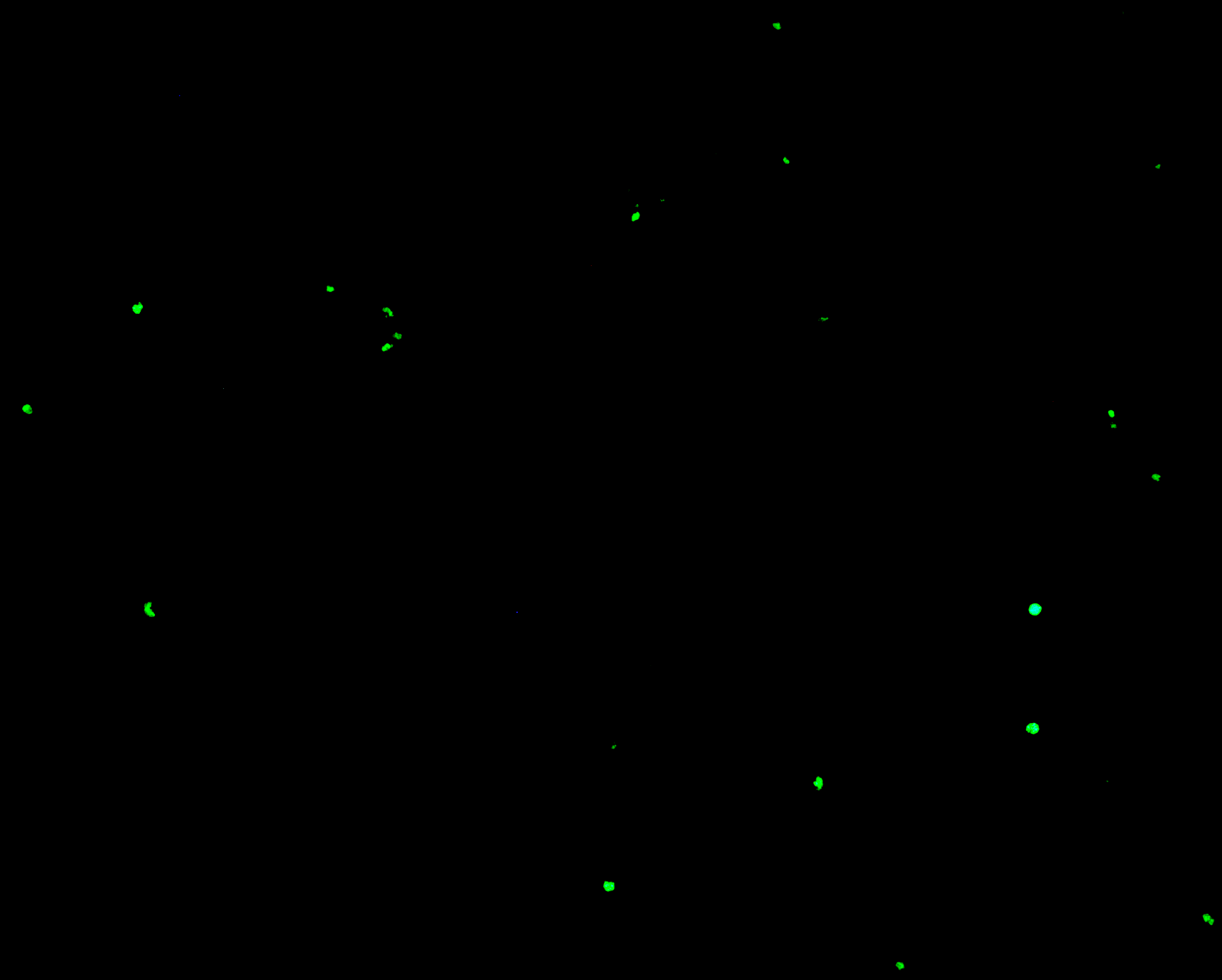

Supplement: Supplementary file 1 [file nutrients-18-00021-s001.zip › Raw image of Fig 2G and 3E/Fig 3E/Fig 3E-5.tif]

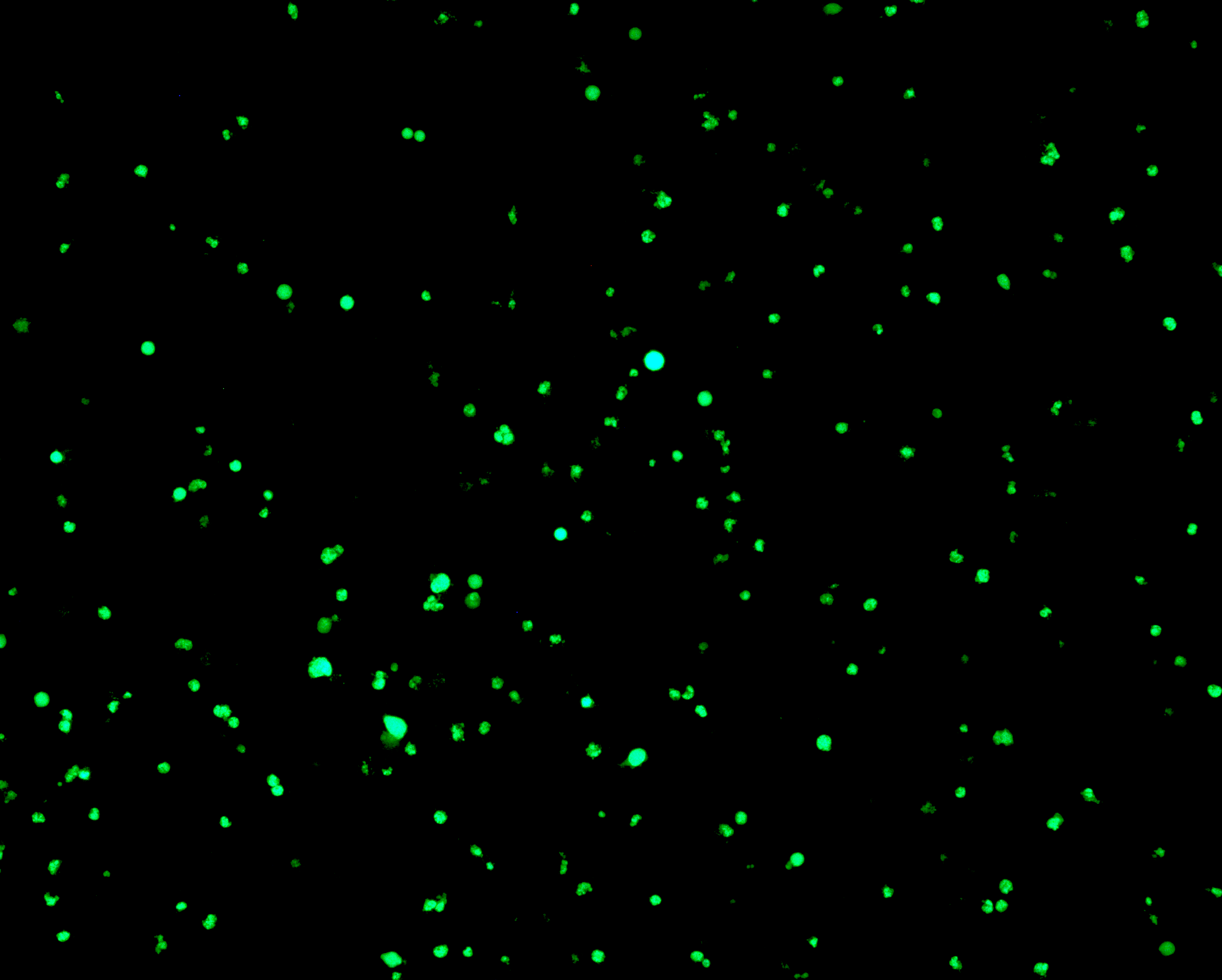

Supplement: Supplementary file 1 [file nutrients-18-00021-s001.zip › Raw image of Fig 2G and 3E/Fig 3E/Fig 3E-6.tif]

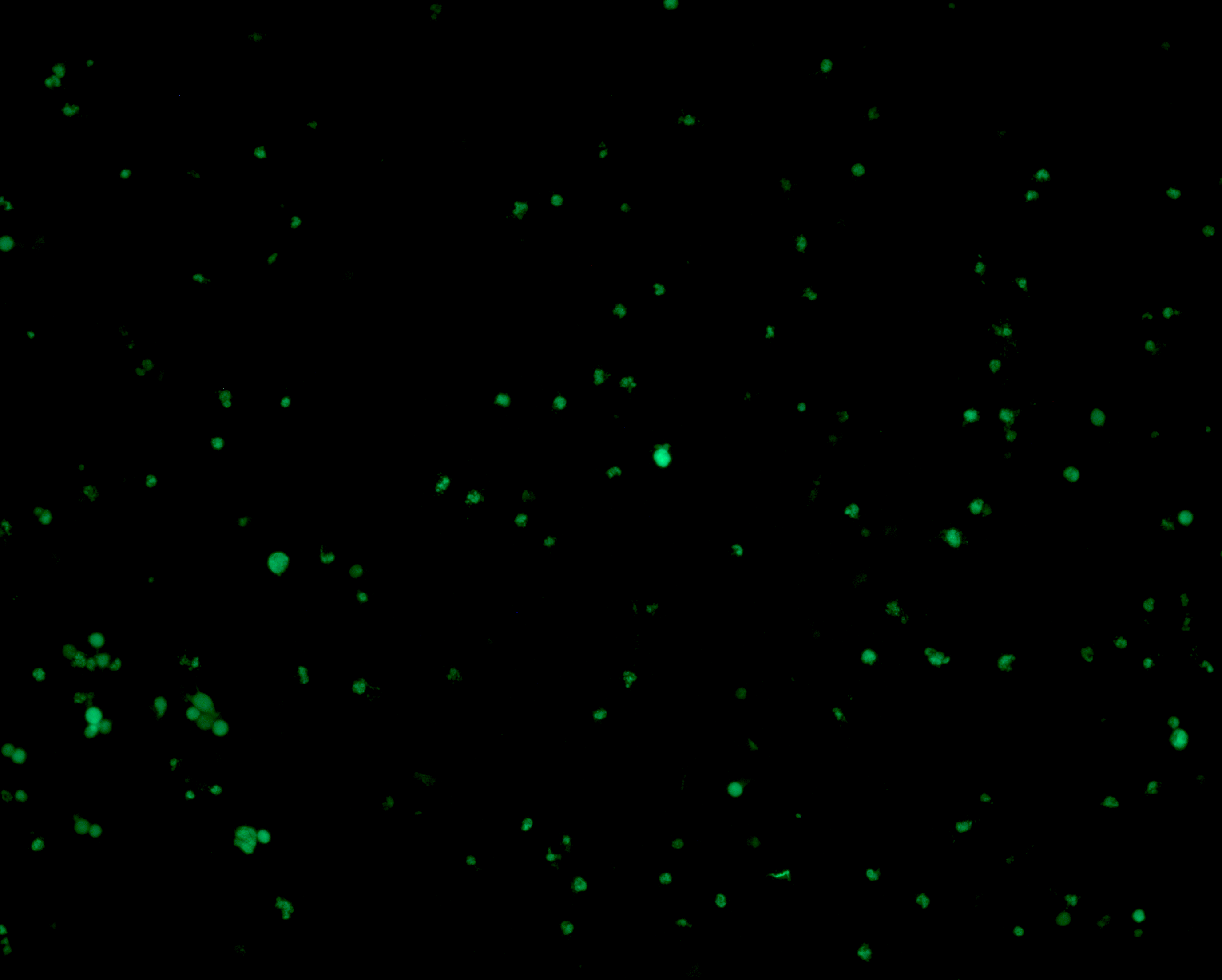

Supplement: Supplementary file 1 [file nutrients-18-00021-s001.zip › Raw image of Fig 2G and 3E/Fig 3E/Fig 3E-7.tif]

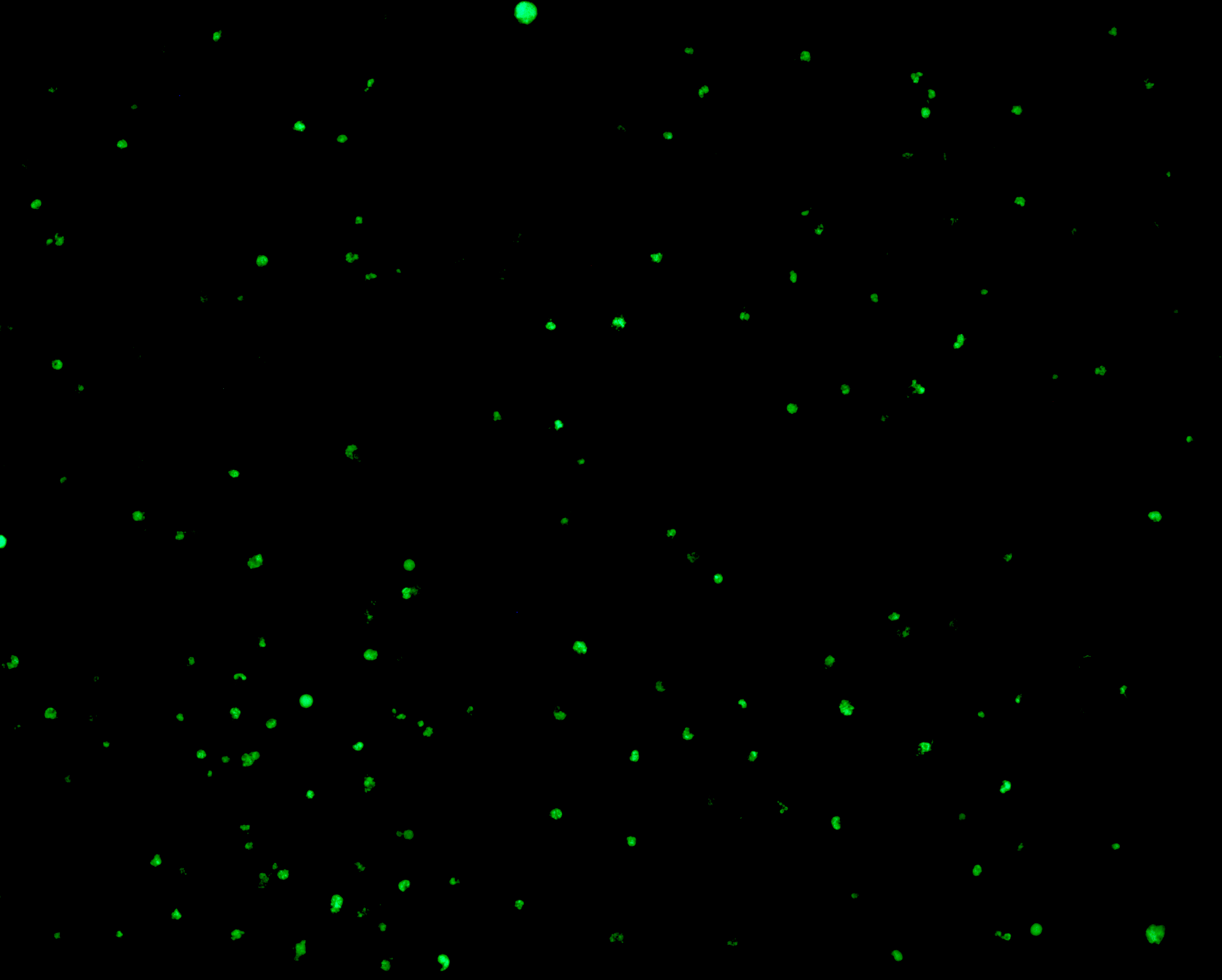

Supplement: Supplementary file 1 [file nutrients-18-00021-s001.zip › Raw image of Fig 2G and 3E/Fig 3E/Fig 3E-8.tif]
